# Neuromodulation by nanozymes and ultrasound during Alzheimer’s disease management

**DOI:** 10.1186/s12951-024-02406-7

**Published:** 2024-03-30

**Authors:** Viswanathan Karthika, Badrinathan Sridharan, Ji Won Nam, Daehun Kim, Hae Gyun Lim

**Affiliations:** 1 Department of Biomedical Engineering, Pukyong National University, Busan, 48513 Republic of Korea; 2 Industry 4.0 Convergence Bionics Engineering, Pukyong National University, Busan, 48513 Republic of Korea

**Keywords:** Alzheimer’s disease, Nanozymes, Ultrasound, Stimuli-responsive, Drug delivery

## Abstract

Alzheimer’s disease (AD) is a neurodegenerative disorder with complex pathogenesis and effective clinical treatment strategies for this disease remain elusive. Interestingly, nanomedicines are under extensive investigation for AD management. Currently, existing redox molecules show highly bioactive property but suffer from instability and high production costs, limiting clinical application for neurological diseases. Compared with natural enzymes, artificial enzymes show high stability, long-lasting catalytic activity, and versatile enzyme-like properties. Further, the selectivity and performance of artificial enzymes can be modulated for neuroinflammation treatments through external stimuli. In this review, we focus on the latest developments of metal, metal oxide, carbon-based and polymer based nanozymes and their catalytic mechanisms. Recent developments in nanozymes for diagnosing and treating AD are emphasized, especially focusing on their potential to regulate pathogenic factors and target sites. Various applications of nanozymes with different stimuli-responsive features were discussed, particularly focusing on nanozymes for treating oxidative stress-related neurological diseases. Noninvasiveness and focused application to deep body regions makes ultrasound (US) an attractive trigger mechanism for nanomedicine. Since a complete cure for AD remains distant, this review outlines the potential of US responsive nanozymes to develop future therapeutic approaches for this chronic neurodegenerative disease and its emergence in AD management.

**Graphical Abstract:**

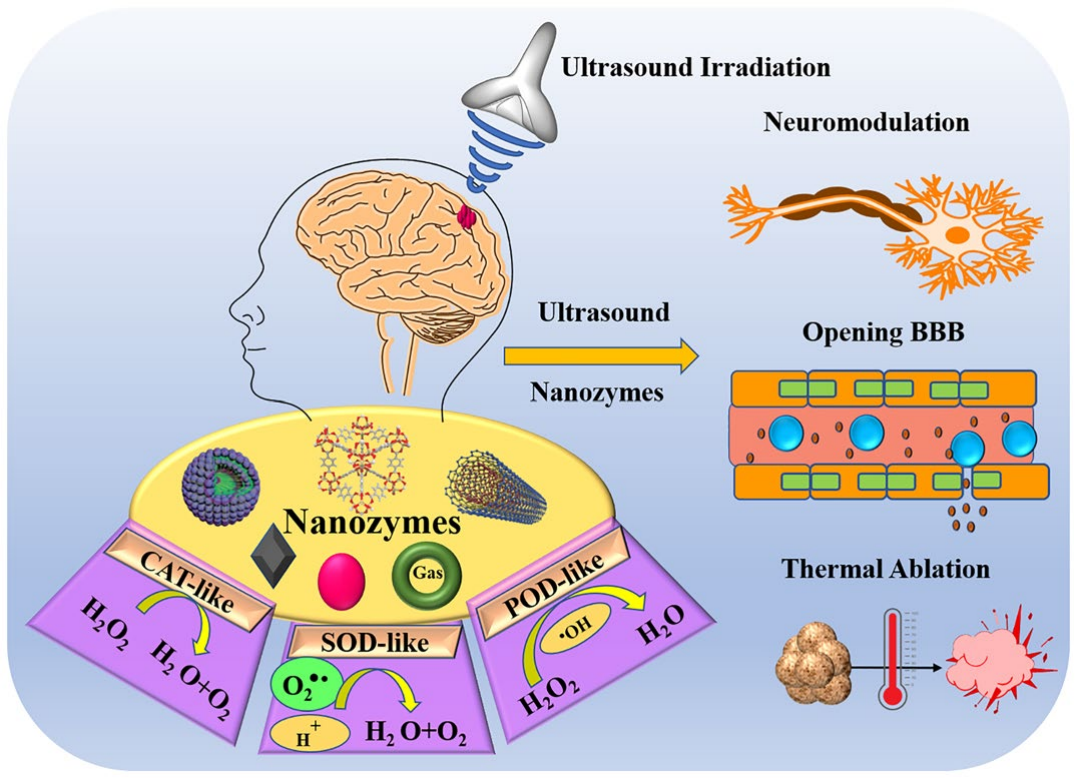

## Introduction

Alzheimer’s disease (AD) is an irreversible, progressive, chronic neurodegenerative disorder that occurs gradually and results in memory loss, unusual behavior, personality changes, and a decline in thinking abilities. AD is named after Dr. Alois Alzheimer, a German doctor who in 1907 noticed changes in the brain tissue of a woman who had died of an unusual mental illness. There was a loss of nerve cells in various regions of the brain that are vital for normal memory and mental abilities [1]. AD accounts for 70% of reported dementia cases. Numerous theories have been debated for years concerning the onset and advancement of AD, including the Aβ peptide/Amyloid cascade theory, the Tau (τ) protein theory, and the cholinergic hypothesis, among others. So far, the Aβ hypothesis has been the dominant explanation for the progression of AD. This theory suggests that the aggregation of Aβ and hyperphosphorylation of τ proteins are central features, triggering neuroinflammation through activated microglia and astrocytes, along with concomitant mechanisms like oxidative stress and apoptosis, which accelerate the advancement of the disease [2].

Currently, there are more than 30 million individuals worldwide grappling with AD, and this number is anticipated to exceed 100 million by 2050. Predictions suggest that in 2050, a new case of AD could arise approximately every 33 seconds, resulting in around a million new cases annually. Despite rigorous research efforts following its initial identification in 1906, the precise molecular foundation of AD underlying pathogenesis remains partially elusive. Additionally, due to its intricate and multifaceted origins, there are no effective interventions available to halt or reverse the progression of AD [3]. The consensus is that AD develops due to a combination of various risk factors, which include old age, family history, genetic predisposition, education, and instances of brain injury [4]. The population of individuals with younger onset AD consists of roughly 200,000 people below the age of 65, while those aged 65 years or older make up to 5 million.

Current pharmacologic treatments (drugs) available are targeted towards managing dementia caused by AD progression, and pertaining destruction of neurons. However, efficacy of those clinically prescribed drugs varies from person to person and their effectiveness are also under constant scrutiny [5]. The U.S. Food and Drug Administration (FDA) has sanctioned five medications for treating AD: rivastigmine, galantamine, donepezil, memantine, and the combination of memantine and donepezil. Aducanumab is the sole treatment with the potential to slow AD progression, but its testing has been limited to individuals with mild cognitive impairment or early-stage Alzheimer’s dementia. Apart from memantine, the remaining drugs temporarily enhance cognitive symptoms by increasing neurotransmitter levels in the brain. Memantine obstructs specific overactive receptors that could harm nerve cells. Nevertheless, all these existing therapeutic approaches (like rivastigmine, galantamine, memantine, and donepezil) seem to address secondary aspects and aren’t directly implicated in downregulating the primary pathogenic factors of AD development [6]. However, traditional medication forms like solid and liquid doses face challenges such as undergoing first-pass metabolism and unfavorable pharmacokinetics. These issues contribute to lower bioavailability and the necessity for higher dosages treatment. Successful oral administration entails effective absorption, sustained systemic circulation, and the capacity to traverse the blood-brain barrier (BBB) to reach the desired target location. The efficacy of drugs is impacted by physicochemical characteristics like solubility, molecular weight, polarity, and partition coefficient. Insufficient responses can lead to treatment inefficacy or failure. Moreover, bioactive substances such as proteins and peptides encounter elevated metabolism when taken orally, leading to suboptimal therapeutic results. Traditional treatments mainly relieve symptoms and temporarily enhance cognitive functions, yet they lack specificity for the brain and can trigger undesired effects [7]. Successful drugs for AD should focus on downregulation of primary pathogenic mechanisms like Aβ accumulation and pertaining events such as microglial activation, neuroinflammation and oxidative stress.

**Fig. 1:**
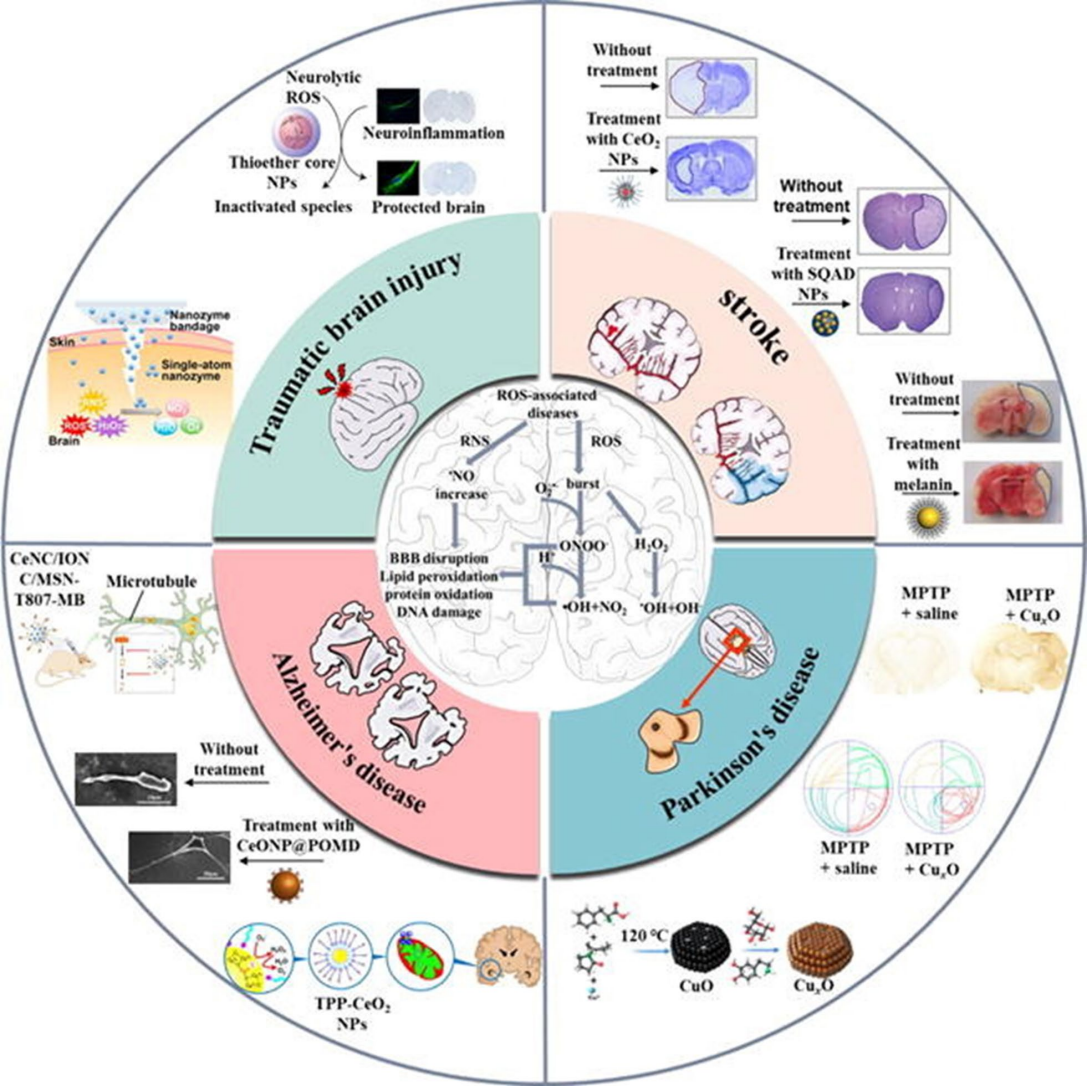
Catalytic nanozymes for central nervous system disease. Reproduced with permission [8]. copyright 2021, Elsevier

Reactive oxygen species (ROS), a by-product of aerobic metabolism, is essential for signal transmission within a specific range. ROS and nitric oxide (NO) play crucial roles in cellular processes but can cause damage when their levels become excessive which makes them a central factor in progression of many metabolic and infectious dieseases [8]. Excessive ROS leads to oxidative stress, damaging cells and tissues. NO, important as a neurotransmitter and neuromodulator, forms harmful ONOO−when overproduced. An excess of reactive oxygen and nitrogen species (RONS) disturbs gut flora balance, triggering sub-digestive stress and is associated with various central nervous system (CNS) diseases. This overabundance of RONS also activates immune responses, further damaging cells and tissues, and compromises the blood-brain barrier, contributing to lipid peroxidation, DNA damage, and neurodegenerative diseases. These CNS conditions, closely linked to RONS (Fig. 1), remain largely incurable, with current treatments only offering relief from the symptoms rather than focusing on the vital cause of the disorder. The exact pathogenesis of these diseases influenced by RONS is still not fully understood despite extensive research. Several cellular enzymes continulsy neutralizes these RONS produced in the cells, but exisiting disease conditions make them inactive or less effiencent in RONS sacavenging [8]. Development of drugs and pertaining formulations is the need of the hour to encounter such key mechanisms for better therapeutic efficacy.

The emerging potential of nanotechnology is ushering in fresh opportunities in the field of diagnosing, treating, and ultimately curing human diseases. The utilization of nanocarriers or nano-formulations for AD therapy brings several advantages, including the ability to bypass hepatic metabolism, lower dosage requirements, improved drug stability, enhanced bioavailability, and precise targeting at the intended treatment site. Successfully delivering drugs to the brain poses a formidable obstacle in the treatment of neurological disorders. The movement of molecules within the brain tissue encounters a formidable physical hurdle, known as the BBB, positioned between the central nervous system CNS and the peripheral circulatory system. This barrier stands as one of the most difficult physiological challenges [9].

**Fig. 2:**
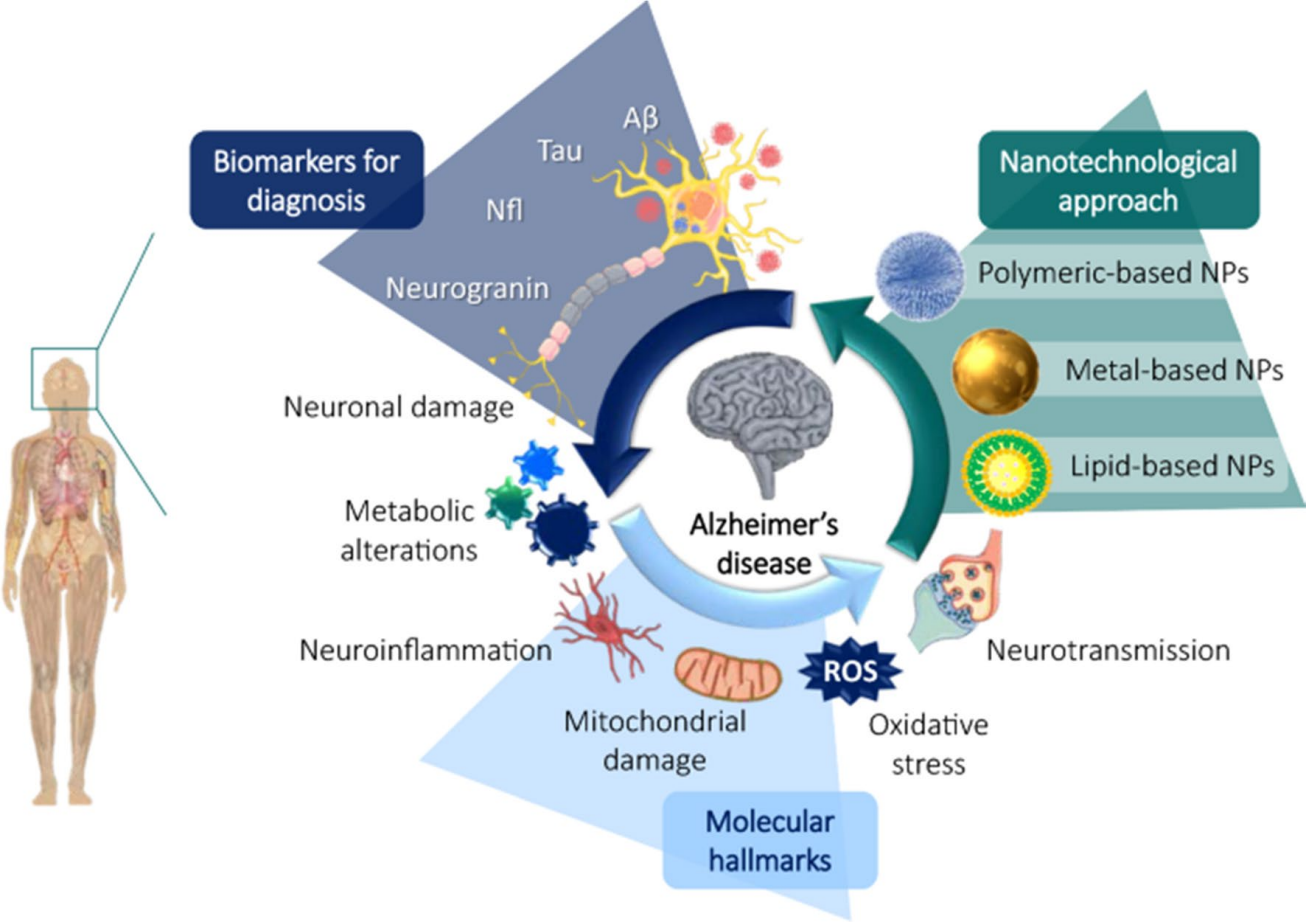
Nanomaterials for Alzheimer’s disease management. Reproduced with permission [10]. copyright 2021, Springer Nature

The emergence of nanomedicine has introduced hopeful remedies for addressing the challenges of the BBB in managing CNS disorders (Fig. 2). Nanomedicine involves the creation of substances and tools that possess dimensions between 1 and 100 nanometers [11]. These structures are made from a diverse range of materials, including biodegradable polymers, lipids, proteins, and organometallic compounds [12]. Metal based nanoparticles (NPs) have proven to be a valuable therapeutic strategy for effectively managing AD through precise drug delivery. Metallic group materials such as gold, silver, selenium, iron, and cerium, all of which possess notable anti-AD characteristics [13]. Among these, gold (Au) NPs stand out due to their capability to pass through brain endothelial cells via transcytosis without requiring surface alterations. Positively charged Au-NPs exhibit excellent permeability across the BBB and possess neuroprotective qualities [14]. By combining with glutathione, Au-NPs have been identified as having an anti-Alzheimer’s effect, demonstrated by their ability to hinder the aggregation of Aβ proteins [15]. One of the most promising and advanced approaches for delivering therapeutic medications precisely at the brain region of AD patients involves utilizing polymeric NPs [16]. These intelligent nanosystems offer a fresh and safe means to enhance the effectiveness of parenterally administered pharmaceuticals by improving their pharmacokinetics and distribution throughout the body. Polymeric NPs are comprised of block co-polymers, constructed from both naturally derived basic units like albumin and polysaccharides, and synthetic materials that are often naturally present within the body [17]. Some researchers have proved polymeric NPs possess high biocompatibility, biodegradability, and easy excretion [16, 18].

The emerging nanocarbon material, a combination of graphene oxide (GO) and graphene, has generated significant interest due to its potential in both diagnosing and treating Aβ-related diseases, primarily focusing on AD. Xiao et al. reported that a neuroprotective peptide linked with graphene quantum dots improved memory and learning in a mouse model of AD [19]. Additionally, Zhang et al. (2020) explored the potential use of nanomaterial-based on GO for targeted delivery and treatment for AD and pertaining conditions. However, the use of such nanomaterials is subject to certain limitations. Research on Aβ has demonstrated that GO can lower levels in HEK293T-APP and SHSY5Y-APP cells. These materials interact with the amyloid precursor protein (APP), leading to the degradation of β-site in APP cleaving enzyme 1. Consequently, the decrease in APP accumulation results in reduced Aβ generation. Furthermore, they interact with endosomes for lysosomal clearance of Aβ peptides. These collective actions effectively lead to a decrease in Aβ-amyloid levels. Moreover, experiments carried out on mouse models involving the suppression of fear memory through tibial fractures under anesthesia have revealed that GO holds potential in reducing Aβ levels and enhancing cognitive function. Particularly in the hippocampal region of 10-month-old mice, GO has shown promise in reducing Aβ levels and improving cognitive performance. These encouraging findings present a novel nanomedicine-based therapeutic approach to address AD [20].

The term “Nanozyme” is a blend of “nanomaterial” and “enzyme, ” following a similar naming convention to that of ribozymes, abzymes, chemzymes, DNAzymes, synzymes, and others. Nanozymes, in comparison to natural enzymes and traditional artificial enzymes, offer benefits like affordability, enhanced stability, scalability in production, and versatile functionalities. These attributes endow nanozymes with greater potential for systematic design and a wider range of applications [21]. The advancement of nanotechnology research could be driven by the encouraging clinical prospects of nanocatalytic process carried out by nanozymes, not only for AD but also for monitoring and treating various other diseases [22]. Nowadays researchers are focusing on nanozymes for AD because of potential application of antioxidant nanozymes in brain disorders [23]. For instance, C-dot superoxide dismutase (SOD) nanozymes were engineered to safeguard neuronal cells in a male mouse model of ischemic stroke (IS) [24]. Additionally, a peptide-guided manganese dioxide nanozyme (PNzyme/MnO_2_) was developed which possesses SOD- and catalase (CAT) like activities [25]. In IS models, this nanozyme displayed potent thrombolytic effects, minimized ischemic brain tissue damage, and suppressed both astrocyte activation and the release of pro-inflammatory cytokines. Compared to NPs, nanozymes play a primary role in AD therapy due to the constant high levels of oxidative stress that brain cells of AD patients experience. Nanozymes generally employ two strategies to address these conditions. Firstly, they directly neutralize excessive reactive oxygen species (ROS) and downregulate the inflammatory signalling [26]. Secondly, they modulate signaling molecules linked to the pathology and facilitate cell communication for therapeutic purposes [27]. Therefore, nanozymes are presumed to be an excellent therapeutic alternative for the management of AD. Though NPs or nanocatalytic formulations are known for its enhanced bioactivity compared to the free drug counterparts, the site specific targetting and adverse effects are still potential road blocks for its way to clinical translation. Thses challenges were encountered with several external stimulus from variety of processes like magentic field, light, sound and so on. Among various external force that aid in enhanced bioactivity of nanomedicine, ultrasound plays a crucial part with significant advantages over the other methods, which will be discussed comprehensively in the following sections.

Ultrasound (US) therapy offers several advantages in the treatment of brain diseases. While it may have limited spatial resolution compared to other external stimuli, it is characterized by non-invasiveness, easy accessibility, robust tissue penetration capability, real-time imaging, and the availability of relatively mature instruments [28]. Unlike some other stimuli like light, magnetic fields, electric fields, and radiation, US therapy minimizes systemic toxicity to adjacent healthy tissues. Light-based photo-stimulation, for instance, is hindered by poor tissue penetration, low extinction coefficient, and limited selectivity. Electric field stimulation can raise local temperatures, while external radiation therapy can damage surrounding healthy tissues, leading to significant toxic side effects [29]. Magnetic field-based therapies, although capable of tissue penetration, may suffer from leakage of magnetic nanomaterials into surrounding normal tissues. In the context of US therapy for brain diseases, the rational design of micro/nanomaterials plays a crucial role. These materials can contain sonosensitizers, enabling the production of ROS under US irradiation for effective tumor cell killing [30]. Focused ultrasound (FUS) can induce microbubble oscillations in blood vessels, temporarily opening the BBB and facilitating localized drug delivery to the brain. US can also remotely trigger "intelligent" nanosystems for controlled and targeted drug release. Some nanomaterials are designed as synergists to enhance the thermal ablation effect of high-intensity focused ultrasound (HIFU) by modifying the tumor's acoustic microenvironment [31].

This review aims to highlight the importance of stimuli-responsive nanozymes in the fields of biology, and medicine with special focus on role of ultrasound in biomedical applications of nanozymes. It primarily focuses on research endeavors dedicated to customizing ultrasound-responsive nanozyme systems, demonstrating responsiveness to single or multiple substrates. Furthermore, the review delves into how nanozymes contribute to advancements in biomedicine, showcasing the underlying design strategies employed to enhance diagnostic and therapeutic effectiveness using ultrasound-responsive nanozymes for various diseases, with a particular emphasis on AD. Ultimately, the review addresses current challenges and future prospects in developing ultrasound-responsive nanozymes and maximizing their theranostic potential.

## Nanomedicine and nanozymes

Considering the current advancements in the field, nanozymes can be characterized as nanomaterials that facilitate the conversion of enzyme substrates to products, adhering to enzymatic kinetics such as the Michaelis–Menten equation, even in physiologically relevant conditions. It’s important to note that the molecular mechanisms of these reactions may vary between nanozymes and their corresponding enzymes. Although the current definition specifies nanozymes working under physiologically relevant conditions, it is highly probable that nanozymes can operate effectively in harsh conditions that would typically denature enzymes. It's crucial to highlight that the field of nanozymes is rapidly evolving, and establishing a universally accepted definition for nanozyme is expected to remain an open question for a considerable period [21].

Nanomedicine has influenced the clinical management of various diseases over the past 2 decades and has shown significant success in treatment of several deadly infections and certain challenging metabolic disorders. In recent years, there has been a growing fascination with nanozymes, which are artificial enzymes that exhibit functions similar to natural enzymes. These nanomaterials with enzymatic functions, have grabbed the attention of experts due to their enhanced stability in harsh conditions, increased durability, and cost-effectiveness compared to their natural counterparts [32]. Numerous nanomaterials with properties of certain enzymes like oxidase (OXD), glucose oxidase (GOD), peroxidase (POD), CAT, SOD, and glutathione peroxidase (GPx) were reported, that could influence the wide range of biological applications [33]. Since the discovery on enzyme like activity of the ferromagnetic NPs by Yan et al. in 2007, scientists and clinicians have directed their attention towards nanozymes and development of numerous variants, to counter a wide array of medical conditions and pertaining challenges. Remarkable progress was observed in the field of nanozymes research leading to development of metal-based materials which include titanium, manganese, iron, copper, zinc, zirconium, palladium, silver, and etc. On the other hand, nanozymes containing composites of carbon, boron and bismuth, which are devoid of metals were also successfully developed [34]. The concept of nanozymes has transformed our fundamental knowledge of biology and chemistry and established a multidisciplinary domain that can open up a wide range of opportunities in biosensing, bioimaging, antibacterial, anti-oxidation, therapeutics, environmental protection, and so on [35].

Nanozymes are predominantly reported to exhibit three main types of oxidoreductase activities, namely antioxidant enzymes and POD, OXD that includes horseradish peroxidase (HRP) and GPx. POD catalysis oxidation of the substrates by utilizing hydrogen peroxide (H_2_O_2_) as an electron acceptor. HRP oxidize various chromogenic substrates such as tetramethylbenzidine (TMB), diaminoazobenzene (DAB) and o-phenylenediamine (OPD), in the presence H_2_O_2_ and this is the key reaction applied for detection of various target molecules in experiments like ELISA, immunofluorescence etc. GPx shows the vital function of inhibiting lipid peroxidation reaction and in turn protect organisms from oxidative damage and pertainin metabolic abnormalities. CAT is also an antioxidant enzyme that can catalyze H_2_O_2_ to produce oxygen (O_2_) and water (H_2_O). SOD is an important antioxidant enzyme in organisms, which can catalyze the conversion of superoxide into O_2_ and H_2_O_2_ [36, 37]. OXD is an enzyme that uses O_2_ as an electron acceptor and can reduce O_2_ to H_2_O and H_2_O_2_. Gox is also an important oxidoreductase, which can catalyze the production of H_2_O_2_ and D-glucono-d-lactone from glucose. Due to its unique catalytic properties, GOx is often used in biological testing [38]. Nanozymes are categorized based on their mode of action towards single or multiple substrates, as well as the physicochemical properties of nanozymes, for applications in biomedicine. Additionally, there are only an few studies that explored the use of ultrasound-in aiding or enhancing the efficiency of nanozymes for therapeutic purposes.

## Single-substrate mechanism in nanozymes

This section, discusses about the single substrate reactions of nanozymes to demonstrate its structure, chemical compositions and catalytic mechanisms. Generally, the enzymatic properties of artificial enzymes were observed at optimum with stable and water-soluble platforms and hance the functional groups that render the enzymatic properties were incorporated as nanoformulation for enabling efficient catalytic reactions. As research progressed, nanomaterials consisting of multivalent metal ions or carbon atoms were utilized as the core in the frameworks for a stable nanozyme with multiple applications. The subtypes of widely reported nanozymes with single-substrate mechanisms were categorized according to reactions they catalyze and the types of natural enzymes they mimic. Typically, nanozymes with a single-substrate mechanism display kinetics of Michaelis–Menten catalytic profile, involving both substrate binding and reaction phases.

### Hydrolase

**Fig. 3:**
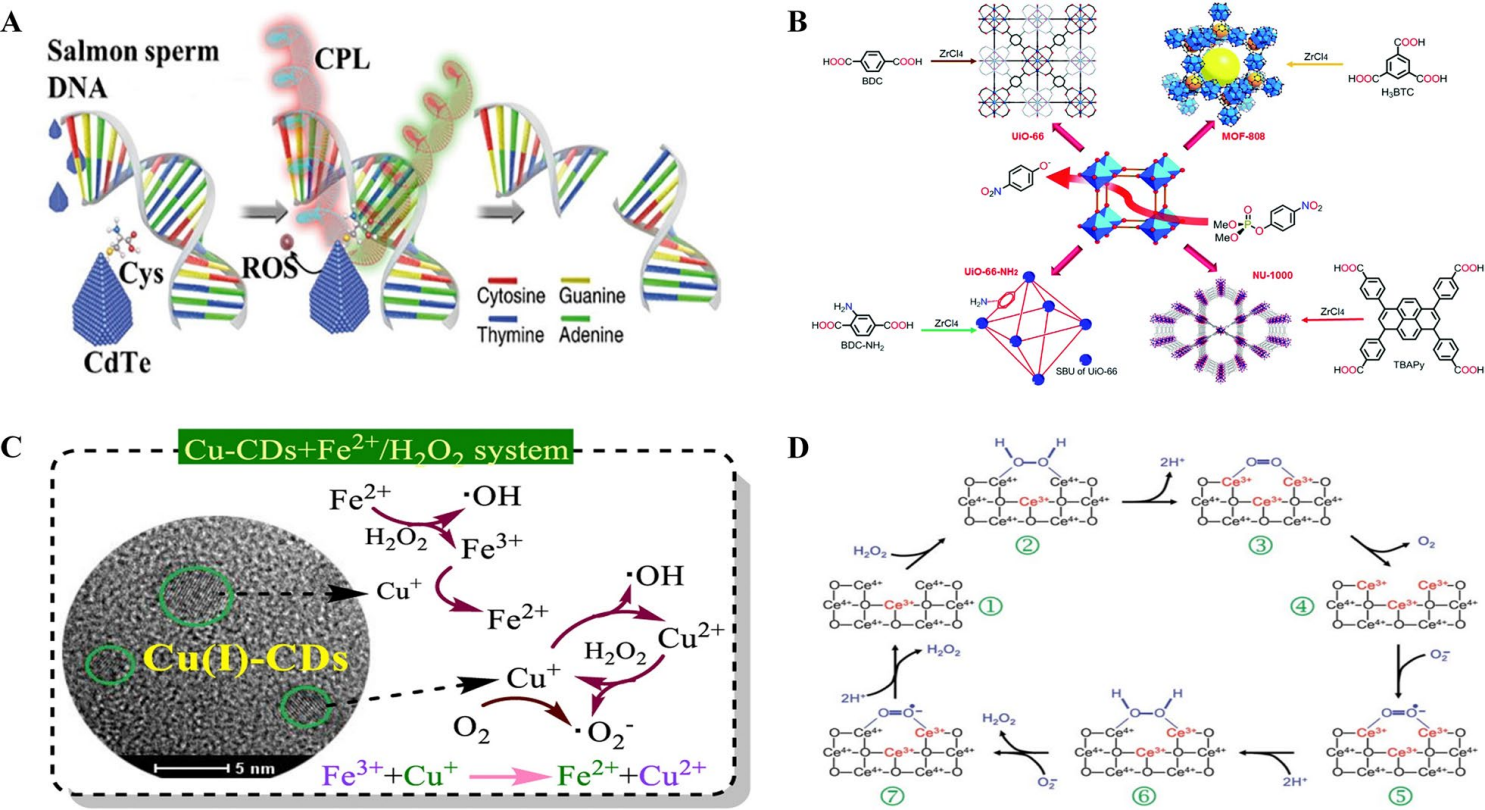
**A** Strategy for site-selective DNA cleavage. Reproduced with permission [39]. copyright 2018, Springer Nature. **B** Illustration of the synthesis and DMNP hydrolysis of PTE mimicking MOFs. Reproduced with permission [40]. copyright 2016, Royal Chemical Society. **C** Schematic illustration of radical generation by different Cu-CDs catalysts. Reproduced with permission [41]. copyright 2020, Elsevier. **D** A model of the reaction mechanism for the oxidation of hydrogen peroxide by nanoceria and the regeneration via reduction by superoxide. Reproduced with permission [42]. copyright 2011, Royal Chemical Society

Jiang and co-workers published that chiral cysteine-modified CdTe NPs has the ability to catalyze restriction endonuclease type reaction for recognition and cleaving of restriction site in DNA double strands specifically that possess more than 90 base pairs. The study clearly reports that the endonuclease mimicking property of nanozymes is the evidence for a non-biological materials that can aid in gene editing and other biological applications at the molecular level (Fig. 3A). The mechanism behind the nuclease type nanozymes, is that the nanomaterial part of the nanozymes acts as the electron donor for ROS generation and DNA acts as the electron acceptor resulting in cleavage of phosphodiester bond [39].

Phosphodiesterase (PDE) is an enzyme that catalyzes the hydrolysis of phosphodiester bonds, aided by multiple metal ion centers from 3D structure of PDEs, collectively and electron donors from the substrate molecules. Another enzyme called phosphotriesterase (PTE) contains metal ion centers for hydrolyzing tri-ester bonds. The active sites of PTE are Zn–OH–Zn moieties, which have been successfully replicated by several catalytic nanomaterials in the form of Metal-Organic Frameworks (MOFs) (Fig. 3B) [40]. Farha, Hupp, and their team reported successful denaturation of chemical warfare agents like nerve agents and their simulants with multiple MOF based nanozymes as artificial PTE. Among the MOFs tested, Zr6-containing NU-1000 with a large pore size (3.1 nm) demonstrated the ability to accelerate the hydrolysis of nerve agent simulants, such as dimethyl 4-nitrophenyl phosphate (DMNP) and GD. The Zr–OH–Zr moieties in the MOF effectively imitated the Zn–OH–Zn catalytic sites found in natural enzymes. Researchers have explored the use of MOFs to replicate the catalytic properties of phosphotriesterases, showing promising applications in denaturing several other chemical warfare agents and potentially contributed to improve environmental protection.

The hydrolysis reactions are vital in various biological processes involving breaking down complex molecules into simpler components. Nanozymes catalyzing hydrolase type of reactions gained significant attention in recent years due to their potential applications in drug delivery, diagnostic assays, and targeted therapy. In case of environmental applications water treatment and pollutant degradation were also successfully achieved by this type of nanozymes. The ease of synthesis, stability, and tunability of their catalytic properties make hydrolase nanozymes promising alternatives to natural enzymes for several practical applications [43].

Hydrolysis of nucleotides by nucleosidases and phosphate group cleavage by phosphatases are some of the best examples of reactions catalyzed by hydrolases. These enzymes play crucial roles in biological systems and environmental protection as they degrade larger molecules into simple units. Although there are limited studies on the catalytic mechanisms of hydrolase-like activity in nanozymes, bond breakage is closely related to free radicals quenching mechanisms [44]. Several nanomaterials have been investigated to mimic hydrolases, and the most notable works are discussed in this section.

### Peroxidase

Peroxidase, a natural enzyme present across a spectrum of organisms including plants, humans, and bacteria, serves a fundamental role. Its primary function involves converting H_2_O_2_, a byproduct of oxygen respiration, into harmless substances. Peroxidase enzymes function as agents that neutralize free radicals (e.g. glutathione peroxidase) and contribute to the body's defense mechanisms against invading pathogens (e.g. myeloperoxidase). Moreover, peroxidases find widespread application in bioanalytical and clinical chemistry contexts, facilitating the detection of analytes through colorimetric assays. Substantial efforts have been directed towards creating effective alternatives to natural peroxidases, known as peroxidase mimetics, to address challenges observed in natural enzymatic peroxidation [45].

Peroxidases belong to a group of enzymes that facilitate the oxidation of substrates using peroxides. In 2007, Yan and his team published the peroxidase-like behavior of magnetite NPs containing various sizes of Fe_3_O_4_. These NPs acted as catalysts, converting 3,3,5,5 TMB into a blue-colored product by reducing H_2_O_2_. Additionally, Fe_3_O_4_ was found to imitate the activity of HRP by inducing the oxidation of DAB into a brown-colored product and OPD into an orange-colored product [46, 47].

### Superoxide dismutase

The imbalanced ROS lead to oxidative harm in biological systems. SOD is the inherent defense mechanism against the superoxide anion O_2_^−^, converting it via dismutation into H_2_O_2_ and O_2_. In order to surpass the constraints of natural SOD and effectively counter oxidative stress, a range of nanomaterials have been investigated to imitate SOD functioning [48]. Based on various successful reports on influence of nanozymes in scavenging O_2_^−^, (e.g., Ce, Pd, MnO_2_, PB, fullerene) they are considered as promising alternatives to natural SOD. Nanozymes possess multiple enzyme activities according to the structure and pH of the environment they are in. At optimal pH surface ions of nanozymes predominantly catalyzes the SOD like activity. While inappropriate pH provides a stronger electrostatic barrier to superoxide radicals that leads to suppression of the catalytic activity.

Up to now, CeO_2_ NPs have been widely recognized as one of the best candidates for superoxide dismutase activity [49]. Different from other trivalent lanthanides, the cerium component can exist in either Ce^3+^ or Ce^4+^. Due to the presence of Ce^3+^ and Ce^4+^ as well as the oxygen vacancies, nanoceria possesses excellent catalytic properties. Nanoceria reduces superoxide, to form H_2_O_2_ and gets oxidized from Ce^3+^ to Ce^4+^. Subsequently, the interaction between Ce^4+^ and H_2_O_2_ helps in renewal of Ce^3+^ and the oxidation of H_2_O_2_ into O_2_. This process presents a sophisticated approach of restoring reduced nanoceria with consecutive removal of both superoxide and hydrogen peroxide, through a sequence of reactions (Fig. 3D) [42]. The balanced ratio of the nanoceria necessitates the reduction of two superoxide molecules for every H_2_O_2_ molecule oxidized [44]. The ratio of Ce^3+^/Ce^4+^ on the surface of nanoceria is significant for the catalytic property and higher ratio could enhance the SOD-like property of nanoceria [50].

### Oxidase

Oxidases facilitate the conversion of an electron-donating substrate to either H_2_O or H_2_O_2_, utilizing oxygen as the electron acceptor. The oxidase enzyme family is categorized based on the substrate's electron-donating component, encompassing amino groups, CH-OH groups (GOx), Ph-OH groups (polyphenol oxidase), sulfur groups (sulfite oxidase, SuOx), and ferrous ions (such as ferroxidase and cytochrome c oxidase) [51]. Among these, nanozymes with oxidase-like properties that target amino groups have been extensively studied. To date, a considerable array of oxidase mimicry has been uncovered, featuring metals and metal oxides like CuO, MnFe_2_O_4_, and Pt@MnO_2_. The formation of intermediates (like singlet oxygen and oxygen superoxide anion) as well as the electron transfer processes have been shown to significantly influence the oxidase-like behavior of these nanozymes [52].

Progress in the field of nanozymes research has also seen significant advancements in emulating the mechanisms of other members within the oxidase family. Taking cues from the study involving MoO_3_ NPs as SuOx mimics, Chen and colleagues devised PEGylated (polyethylene glycol) MoO_3_−x NPs (P‐MoO_3_−x NPs), capable of catalyzing sulfite oxidation [53]. The process involved the conversion of sulfite to sulfate through two-electron oxidative hydroxylation. This was succeeded by the reduction of [Fe(CN)6]^3−^, enabling the sequential transfer of a single electron to the Mo^V^ intermediate, thereby stabilizing the inactive Mo^IV^ state [53].

### Catalase

Catalase is a typical biological catalyst virtually found in all living systems. It facilitates the decomposition of H_2_O_2_ into H_2_O and O_2_ [24]. The inherent CAT enzyme consists of four iron-containing heme groups, which contribute to its strong interaction with H_2_O_2_. To replicate CAT enzymatic function, a range of metal-containing carbon-based nanomaterials have been engineered. Dan and colleagues prepared ultrasmall gold nanoclusters loaded with indocyanine green (Au NCs-ICG), which demonstrated a catalytic capacity similar to CAT, distinct from POD or SOD. Notably, Au NCs-ICG exhibited significant substrate affinity (Km ≈ 2.02 mM) and exceptional CAT-like performance (Vmax ≈ 4.55×10−3 mMs^−1^) [54].

## Nanozymes with a multi-substrate mechanism

Significant developments in nanotechnology and the understanding of artificial enzymes lead to rigorous exploration of the multiple enzyme-like activities of nanozymes. In this segment, we outline notable nanozymes that exhibit activity with multiple substrates or display varying catalytic reactions within diverse conditions, encompassing varying pH levels, concentrations of glutathione or hydrogen peroxide, and oxygenation states. Physicochemical aspects can significantly impact the performance of nanozymes, particularly within biological environments like disease sites such as cancer [35].

The catalytic activity of multi-substrate nanozymes is often based on their surface properties, which can be engineered to interact with different substrates, facilitating specific reactions. Researchers have been exploring various types of nanozymes with multi-substrate catalytic abilities, such as metal-based NPs (e.g., gold, silver, platinum), metal oxides (e.g., manganese oxide, cerium oxide), and carbon-based nanomaterials (e.g., carbon nanotubes, graphene) [55]. The ability to have multi-substrate catalytic activity makes these nanozymes versatile tools for various applications in different fields, including environmental monitoring, water purification, drug delivery, and disease diagnosis. However, it is essential to carefully design and control the properties of these nanozymes to ensure their selectivity, stability, and biocompatibility in specific applications.

Researchers are investigating the potential of inorganic nanozymes that possess multiple enzyme-like functions as a promising approach for managing cellular metabolic processes [56]. Superoxide anions and hydrogen peroxides are the key reactive oxygen species that control cellular redox status. Several core-shell nanocomposites made from diverse materials have been developed with peroxidase and oxidase enzymes functions at pH 4.0 [57]. Several physico-chemical and biological factors exert an influence on the performance of multifunctional nanozymes and experts focus on addressing these challenges to attain the level of effectiveness for the nanozymes on par with their natural counterparts. Stuti Bhagat and colleagues reported core-shell nanostructures based on Au and CeO_2_, remained stable in liquid suspension and displayed enzyme-like actions similar to POD, CAT, and SOD. The kinetics of the POD like reaction catalyzed by Au/CeO_2_ based core-shell nanozyme’s showed that its efficiency is comparable to natural HRP enzyme. Au/CeO_2_ NPs is considered as the best among the nanozymes with peroxidase activity that showed efficient electron transfer and scavenging of .OH radicals. This core-shell NPs has retained its activity at wide range of pH (from acidic to alkaline) and at extreme temperature (up to 90 °C) which signifies the superiority of Au/CeO_2_ NPs over natural enzymes. Additionally, core-shell NPs, with its POD activity, were efficiently utilized for glucose detection at a range from 100 µM to 1 mM. The proximity of redox potentials between Au^+^/Au and Ce (III)/Ce (IV) is postulated to create a redox pair that enhances the multi-enzyme performance of the core-shell NPs greatly dependent on the redox potentials of Au^+^/Au and Ce^3+^/Ce^4+^. Further, the core-shell nanostructures can also aid in development and advancements in biosensing applications [58].

In comparison to their catalytic activity, the catalytic selectivity of nanozymes is more important for their efficacy. For example, pathogenesis of neurodegenerative diseases are closely linked to free radicals like ROS and reactive nitrogen species (RNS), that indicates the importance of catalytic selectivity to POD like activity in influencing disease management. Various types of NPs have demonstrated proficient multi-enzyme-like capabilities, including POD, CAT and SOD dismutase activities, while they are reported to selectively inhibit the hydroxyl (.OH) generation.

Metal nanozymes with its robustness are considered to be stable under a range of physiological conditions and render efficient bioactivity. Conditions such as AD, Parkinson's disease (PD), traumatic brain injury (TBI), and ischemic stroke (IS) play significant roles in the worldwide incidence of dementia and mortality. The reduction of ROS and RNS levels are considered as primary target mechanism for neuroprotection because they lead to oxidative and nitrosative stress that play a vital role in the progression of these brain-related disorders [59]. The catalytic activity in the physiological environment can be effective when the nanozyme has optimal activity at pH 7.4. Xiaoyu Mu developed trimetallic nanozyme with PtPdMo through a basic method, which is preferentially effective at neutral environment. This nanozyme platforms possess remarkable catalytic activity and environmental specificity. ROS and RNS were effectively scavenged by the catalytic activity of triM system. Importantly, the triM nanozyme favors neutral environments in counteracting OH, ^1^O_2_, and NO radicals, which exhibits its catalytic selectivity [60]. The electron capture mechanism of triM and high affinity for free radicals within neutral environments was explained well by Density functional theory (DFT) calculations. *In vitro* experiments pertaining to neuroprotection through restoration of SOD activity, reduction of lipid peroxidation and downregulation of neuroinflammation *In vivo* that helps in improved cognition, collectively highlight the ability of triM nanozyme in neuromodulation. This investigation introduces a possibility for enhancing nanozyme selectivity in physiological settings and maximizing its applications in brain science. The development of drugs and drug formulations capable of crossing the BBB and achieving target specificity for enhanced activity is essential, and according to the consistently successful reports on neuromodulation potential of nanozymes, it is assumed to play major role in multifaceted challenges posed by brain injuries.

Acute Kidney Injury (AKI) affects renal cells, leading to rapid reduction in renal function, accumulation of waste products in the systems, and disruptions in the balance of water, electrolytes, and acid-base levels. The progression of AKI involves damaging mechanisms like oxidative stress, apoptosis, and inflammation that are the primary targets for a successful treatment of AKI. Nanozymes, possessing enzyme-like characteristics, have grabbed attention for their capacity to scavenge ROS, as well as their stability in variable conditions and biocompatibility. Ultrasmall nanoparticles are safer for the kidney environment due to its size, however, larger nanozymes might pose burden due to inability to get filtered and cause renal toxicity. Zhou Liu and colleagues developed ultrasmall RuO_2_ containing nanozymes as an alternative for catalase, peroxidase, superoxide dismutase, and glutathione peroxidase activities that exhibit remarkable antioxidant properties by mitigating ROS induced apoptosis with minimal toxicity. *In-vivo* administration, RuO_2_ has aided in inhibition of AKI effectively without causing chronic side effects. These evidence on ultrasmall RuO_2_ NPs display its potential utility in management of disorders with ROS induced pathogenic mechanisms, such as atherosclerosis and neurodegenerative conditions [61].

## Physical and chemical factors influencing efficacy of nanozymes and nanocatalysis

Nanozymes and nanocatalysis refer to the use of nanomaterials with enzyme-like or catalytic activities for various applications, ranging from biomedical to environmental and industrial processes. The efficacy of nanozymes is influenced by a combination of physical and chemical factors [50]. Here are some key factors that play a vital role in nanocatalytic processes.

### Effect of physical Factors

Physically, size and surface area are crucial; smaller nanoparticles often have higher surface area-to-volume ratios, boosting catalytic activity due to increased reactant interactions. The shape of the nanoparticles, particularly anisotropic shapes, can significantly impact catalytic performance, with different activities along various crystallographic directions. Surface charge plays a role in electrostatic interactions with substrates, affecting binding and specificity in catalysis. The aggregation state, specifically the degree of dispersion, influences catalytic efficiency, with well-dispersed particles having better reactant access. Porosity, or the internal structure, affects reactant and product diffusion, thus influencing overall efficiency [62, 63].

The catalytic performance of nanomaterials, or nanozymes, is significantly influenced by their size and shape. Studies have shown that smaller nanoparticles generally exhibit higher catalytic activity due to their increased surface-to-volume ratio, which enhances interaction with substrates. For instance, Fe_3_O_4_ NPs with hydrodynamic size of 30 nm, had greater peroxidase-like properties compared to larger 150 and 300 nm particles [64]. Similarly, Fan et al. found that the glucose oxidase-like activity of AuNPs decreased as their size increased, illustrating size-dependent catalytic performance [50]. The shape and morphology of nanozymes also play a crucial role in their biocatalytic performance. Yin and Chen’s groups fabricated Pd nanomaterials in different shapes (nanocubes and octahedrons) and found that Pd octahedrons, with lower surface energy, possessed higher SOD- and CAT-like properties and ROS removal ability compared to Pd nanocubes [65]. Similarly, Pathak and colleagues observed that truncated octahedron-shaped Fe_3_O_4_ nanoparticles had a higher catalytic ability than their spherical counterparts, likely due to the differences in surface energy facets [66]. These findings highlight the potential of nanozymes in combating oxidative stress and suggest that manipulating the size, shape, and morphology of nanozymes can effectively tune their catalytic properties for specific applications [67].

### Effect of chemical factors

Chemically, the composition, such as the choice between metallic, metal oxide, or organic nanomaterials, determines the reaction types which nanozymes can catalyze. Surface chemistry, including functional groups, can enhance activity or selectivity, acting as catalytic sites. The redox potential is crucial in electron transfer reactions. Chemical stability under reaction conditions is essential for sustained activity, with surface modifications often enhancing stability. Nanozymes mimic natural enzymes’ catalytic mechanisms, and understanding these can improve efficacy. Substrate specificity, influenced by binding affinity, affects catalytic efficiency for targeted applications. Environmental conditions like pH and temperature significantly impact activity, with some nanozymes optimized for specific conditions. Co-catalysts or co-factors can synergistically enhance performance [50]. Optimizing these physical and chemical factors is key for tailoring nanozymes for specific applications in fields like medicine, environmental remediation, and industrial processes, with ongoing research aimed at improving their efficacy and versatility.

Numerous reactions occur on the surface of nanozymes. Applying additional coatings or modifying nanozymes can alter their activity through changes in surface charge, microenvironment, and the exposure of active sites. Modifications typically involve addition of small molecules, ions, and polymers, primarily through physical adsorption. In some cases, covalent modifications are also used [62]. Cysteamine-modified, positively-charged Au nanozymes exhibit higher POD-like activity towards TMB substrates, challenging the expected affinity theory due to TMB's positive charge [38]. However, different studies show varying results such as unmodified Au nanozymes and those with negatively-charged coatings like citrate or mercaptoacetic acid sometimes display greater catalytic activity than positively-charged amino-coated ones [68]. The catalytic efficiency is influenced by surface modifications, which can block active sites and reduce activity, as demonstrated by the correlation between the proportion of exposed active Au (I) sites and POD-like activity in variously modified Au nanozymes [69].

### Effect of surface area

In biomedical applications, the therapeutic effectiveness of nanozymes is greatly influenced by their surface area and size [70]. The surface area of nanozymes plays a critical role in several ways such as boosting catalytic activity by offering additional reaction sites for degrading harmful substances or creating therapeutic agents, enhancing the capacity for drug loading, thus improving therapeutic effectiveness, enabling surface modifications, which enhance biocompatibility and aid in specific drug delivery. Further, increase in cellular uptake through improved membrane interactions, improvement in effectiveness of nanozyme-based sensors in diagnostics and imaging; and the degradation rate was altered, which makes surface arrea of a materials, a relatively important influencing factor for the release of therapeutic substances [71]. These aspects collectively enhance the overall therapeutic efficacy of nanozymes. Size, on the other hand, significantly affects the distribution and pharmacokinetic properties of nanozymes, including their circulation time and rate of clearance from the body. Smaller nanozymes tend to be more effective in penetrating cells, thus boosting therapeutic efficiency. The size of nanozymes is crucial in optimizing the Enhanced Permeability and Retention (EPR) effect for targeted drug delivery, especially to cancerous tissues [72]. Additionally, the tissue penetration ability and drug loading capacity of nanozymes are dependent on their size, with smaller nanozymes typically exhibiting a greater surface area-to-volume ratio, facilitating more efficient drug loading. The immune system’s reaction to nanozymes varies with their size, influencing their lifespan in the blood. In preclinical imaging applications, nanozyme size affects both the resolution and signal intensity, determining their suitability for specific diagnostic tasks. Optimizing the size of nanozymes is key for ensuring their biocompatibility and safety, as well as for controlling drug release [73]. Furthermore, the catalytic process of nanozymes is size-dependent, with smaller nanozymes generally possessing more active sites, leading to improved therapeutic results. In summary, adjusting the surface area and size of nanozymes is vital for maximizing their performance in a range of therapeutic applications, including drug delivery, imaging, and catalysis. Researchers are focused on balancing various factors such as biodistribution, cellular absorption, and immune system response to develop nanozymes tailored for specific therapeutic needs.

## Stimuli responsive nanozymes

Stimuli-responsive nanozymes are a fascinating class of nanomaterials that exhibit enzyme-like catalytic activity and can respond to specific external stimuli. These nanomaterials are designed to mimic the behavior of natural enzymes but possess several advantages over their biological counterparts, such as enhanced stability, tunable catalytic properties, and the ability to be activated or modulated in response to different external triggers. They have continuously been a vital part of many important biomedical and environmental applications.

Stimuli-responsive nanozymes can alter their catalytic activity or behavior in response to specific external stimuli which can be physical (e.g., temperature, light, magnetic fields), chemical (e.g., pH, redox potential), or biological (e.g., specific biomolecules or enzymes). By tuning the nanozymes' responsiveness, they can be activated or inactivated on demand, and this makes them highly controllable and versatile.

### pH sensitive

**Fig. 4:**
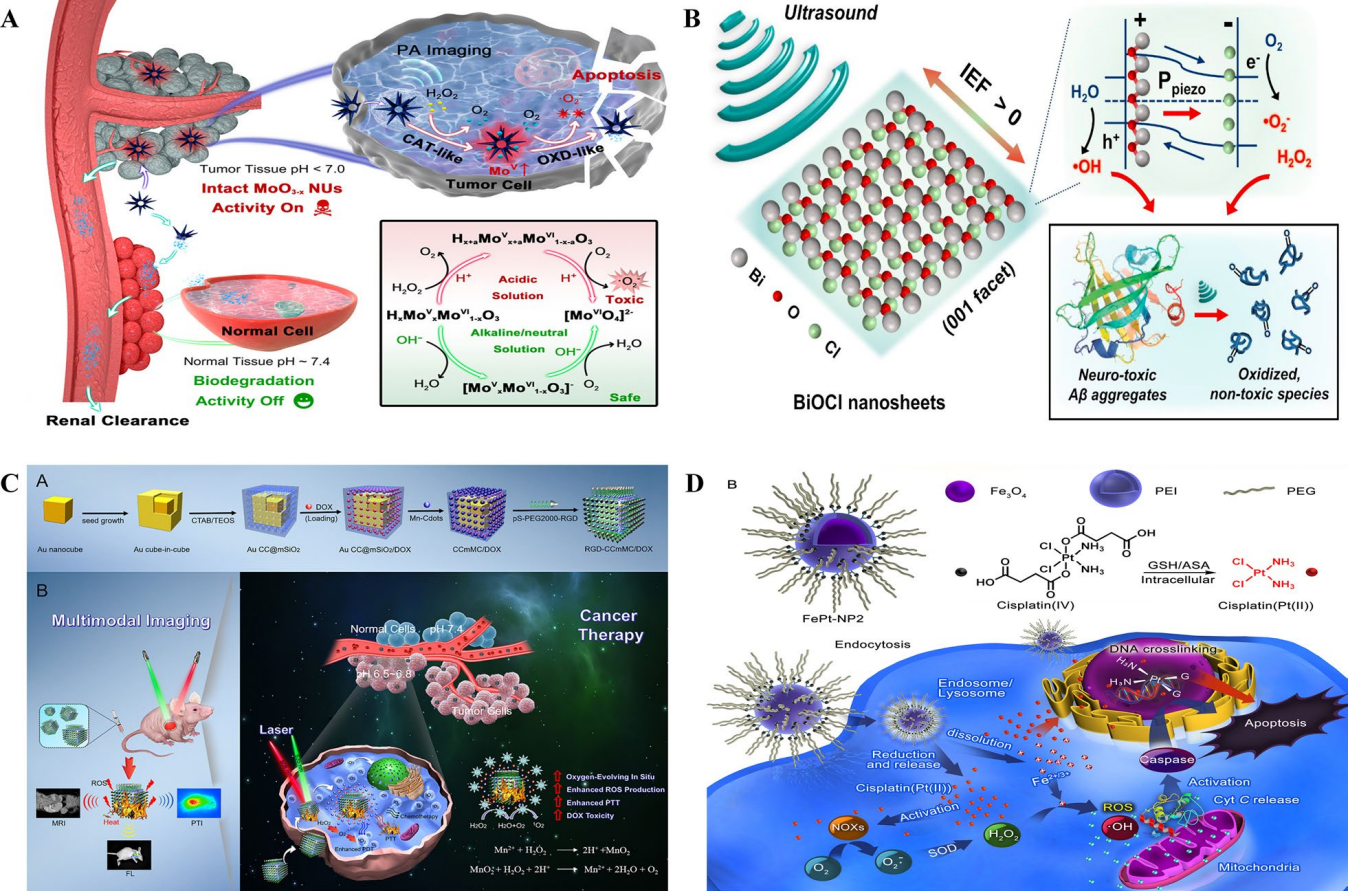
Different stimuli-responsive nanozymes. **A** Schematic Illustration of Biodegradation-Medicated Enzymatic Activity-Tunable Molybdenum Oxide Nanourchins (MoO_3_–x NUs) with the Highly Specific Toxicity to Tumor Tissues via a Multienzyme Stepwise Cascade Catalysis in Acidic Tumor Microenvironment, While Leaving Normal Tissues Unharmed Due to Their pH-Responsive Biodegradation and Subsequent Renal Excretion in Physiological Environment. Reproduced with permission [74]. copyright 2019, American Chemical Society. **B** Schematic illustration of piezoelectric dissociation of Alzheimer's β-amyloid (Aβ) aggregates on the surface of bismuth oxychloride (BiOCl) nanosheets. BiOCl nanosheet is a (001)-facet dominant piezoelectric material that possesses an anisotropic layered structure consisting of [Bi_2_O_2_]^2+^ slabs and interleaved double chloride anionic slabs along [001] direction. Under ultrasound stimulation, the change of local dipole moment in the BiOCl nanosheets induces piezoelectric polarization (Ppiezo) and separates electron–hole pairs by generating internal electric field (IEF). The separated charge carriers trigger piezocatalytic redox reactions of water and dissolved oxygen molecules. The produced reactive oxidative species, such as OH, O_2_^−^, and H_2_O_2_, oxidize and dissociate the highly-stable, self-assembled Aβ aggregates (e.g., Aβ fibrils, plaques) into denatured fragments. Reproduced with permission [75]. copyright 2020, Elsevier. **C,** **A** Schematic illustration of the synthesis process for the versatile RGD-CCmMC/DOX nanovehicles and **B** schematic illustration of the therapeutic mechanism of the RGD-CCmMC/DOX nanoplatforms to enhance the overall anticancer efficiency of triple-combination photodynamic/photothermal/chemo-therapy in a solid tumor. Reproduced with permission [76]. copyright 2019, American Chemical Society. **D** Construction of self-sacrificing iron oxide nanoparticles with cisplatin (IV) prodrug (FePt-NP2) circumvents the endocytosis of cisplatin into the cells. In this way, excess OH are formed, which results in fast lipid and protein oxidation and DNA damage, as well as apoptosis via the ROS/Cyt C/caspase-3 pathway. Reproduced with permission [77]. copyright 2017, American Chemical Society

pH sensitive nanozymes are designed to modulate their catalytic behavior or properties in response to alterations in the pH of their surroundings. One of the most common internal stimuli is the pH value and it is an essential parameter in many biological and environmental systems. pH-sensitive nanozymes have gained considerable attention due to their multifaceted activity and functional stability at wide range of pH that can aid them to render its function according to environment they are in. Acidic pH is an important endogenous signal at tumor microenvironment (TME) and bacterial infection sites. Hu and colleagues introduced a novel concept involving pH-responsive nanozymes called molybdenum oxide nano urchins (MoO_3_−x NUs). These nano urchins displayed a sequential cascade of CAT-like and OXD-like actions, presenting a targeted approach to cancer cells and delivering therapeutic effects within TME. When exposed to acidic conditions, the MoO_3_−x NUs were activated, demonstrating robust catalytic activity against ROS through a H_2_O_2_ → oxygen (O_2_) → superoxide radical (O_2_−) cascade reaction (Fig. 4A) [74]. Nonetheless, their catalytic capabilities vanished in neutral and alkaline surroundings, owing to the oxidation and pH-responsive biodegradation of the MoO_3_−x NUs active MoV components. The application of pH-sensitive biodegradation serves as a remarkable instance of accomplishing switchable enzyme-like behavior. However, Mo-based nanozymes experience irreversible degradation under unfavorable pH conditions [78].

### Sono sensitive

Sonodynamic therapy (SDT) uses ultrasound as extrinsic physical trigger that helps in ROS generation at the microenvironment, they are in through sonochemical reactions. SDT was considered to have an edge over the photo/thermodynamic therapy due to the high tissue penetrability of US [79]. Apart from mechanical and thermal effects of US like pressure oscillations and raise in temperature which are the outcomes during the US tissues interaction, a phenomenon referred to as inertial cavitation characterized by rapid growth and collapse of gaseous cavities, which was also well established and applied in various applications of US platform [80]. ROS generated during cavitation has the ability to induce cancer cell death, and this chemical effect induced by US can be further enhanced in the presence of various organic molecules such as PpIX, hematoporphyrin (HP), Rose Bengal, and etc., which are collectively termed as sonosensitizers [81]. SDT can be generalized as “sonocatalytic” process, in which US provides extrinsic energy input to activate sonosensitizers for catalyzing the ROS generation [82].

Till now, the precise reaction mechanisms involving specific sonosensitizers are actively being investigated. Nonetheless, a growing body of experimental evidence has illustrated that sonosensitizers exhibit a catalytic nature when triggered by US, leading to consistent generation of ROS and aiding in SDT. Early investigations into SDT predominantly concentrated on showcasing the US-triggered ROS production effect exhibited by diverse organic compounds. Nevertheless, these sonosensitizers face challenges due to their limited stability within biological environments, impeding their further application in oncology. To overcome these limitations and enhance SDT effectiveness, nanotechnology has been employed. This includes the use of nano delivery systems or the development of inorganic nanocatalysts as novel sonosensitizers. These approaches aim to improve chemical stability and facilitate the accumulation of these agents within tumors.

Recent investigations have explored the potential of coupling organic sonosensitizers with nanocarriers, as well as exploring the utility of inorganic nano sonosensitizers for sonodynamic applications. Building upon the advancements made in photodynamic therapy (PDT) and photocatalysis, various inorganic photocatalysts have demonstrated their responsiveness to US exposure, resulting in the generation of ROS within aqueous environments. The emergence of TiO_2_ NPs, following their recent demonstration of US-triggered ROS generation capability, has spurred substantial efforts aimed at enhancing the effectiveness of antitumor strategies through SDT. Yuan and colleagues reported that TiO_2_, when present in an aqueous setting, effectively inhibits tumor growth post US treatment [83].

Jang et al. reported on the novel use of piezoelectric bismuth oxychloride (BiOCl) nanosheets in the treatment of AD [75]. These nanosheets can evoke electrochemical reactions by transferring charge carriers to reactants when subjected to mechanical stimuli, such as ultrasound. This property is particularly significant in addressing the accumulation of β-amyloid (Aβ) aggregates, including fibrils and plaques, which is a major pathological hallmark of AD. Clearing these Aβ aggregates is challenging due to their robust β-sheet structure. BiOCl nanosheets, known for their biocompatibility and piezocatalytic activity, effectively disassemble Aβ fibrils under ultrasound stimulation (Fig. 4B). The sono-activated BiOCl nanosheets generate piezo-induced oxidative stress, destabilizing the β-sheets in Aβ fibrils. *In vitro* studies have shown that these sono-activated nanosheets can alleviate the neurotoxicity of Aβ fibrils. Furthermore, *ex vivo* evaluations demonstrated a significant reduction in Aβ plaques in AD mouse brain slices treated with sono-activated BiOCl nanosheets. The piezocatalytic capacity of BiOCl nanosheets allows them to dissociate the β-sheet-rich Aβ fibrils into smaller fragments through the generation of reactive oxidative species under ultrasound. The density of Aβ plaques in AD mouse brain slices was drastically reduced after treatment, indicating the effectiveness of this approach. This study highlights the potential of ultrasound-responsive BiOCl nanosheets in developing piezocatalytic and sonodynamic therapies for AD treatment. It suggests a promising new avenue for addressing a critical aspect of AD pathology by leveraging the unique properties of piezoelectric materials.

### Photothermal sensitive

To explore the impact of photothermal effect on nanocatalytic therapy, Liu et al. performed surface engineering of non-metallic atom doping and laser irradiation to analyze the catalytic performance and its contributing factors. TiN based nanozyme formulation was reported for its POD like activity, which was improved after nitrogen doping. However, enzymatic activity of this material could be enhanced much more with laser irradiation because of its NIR absorbing property and photothermal effect. This led the group of researchers for development of TiN-coated liposomes connecting PEG-modified GOD with pH-responsive manners in conjuncture with tumor micro environment (TME) and can also be triggered by laser irradiation. The integration of multiple mechanisms achieved by GOD co-loading for self-supply of H_2_O_2_, nitrogen doping, and the photoresponsive property, TiN NPs exhibited satisfying anti-tumor potential *In vivo* with minimal side effects [84].

Zhang et al. had fabricated mesoporous silica coated gold cube-in-cubes loaded with doxorubicin (DOX) and anchored by Mn-Cdots. This core/shell nanocomposite was then conjugated with RGD targeting ligands (denoted as RGD-CCmMC/DOX) to achieve the better tumor targeting effect. Accumulation of the nanocomposite at the tumor sites leads to Mn-Cdots efficiently consumed endogenous H_2_O_2_ produced by tumors causing an elevation in oxygen concentrations at TME (Fig. 4C). This helps in efficient attenuation of tumor hypoxia and improved PDT efficacy. This potentially eliminates the concomitant chemotherapeutic drug-resistance caused by hypoxia. Moreover, the gold cube-in-cube core in the nanoplatform has proven to be an advantageous over other photothermal agent, with a 65.6% photothermal conversion efficiency under 808 nm irradiation, which is favourable for anti-tumor therapy. In addition, Mn-Cdots acted as gatekeepers to precisely release the encapsulated DOX in a heat- and pH-sensitive manner at specific-tumor sites. Meanwhile, the nanoplatform serves as a theranostic agent by furnishing complementary information for accurate diagnosis and imaging-guided cancer therapy through FL/PTI/MRI multimodal bioimaging. This work succeeded in designing a versatile nano-platform with the TME-modulating effect, which not only combined PTT, PDT, and chemotherapy for targeted cancer therapy but also forms the basis for designing nanosystems with similar mechanistic approach in cancer treatment [76].

### ROS responsive

Reactive oxygen species (ROS) that includes superoxide anion (O_2_−), hydroxyl radical (·OH), and hydrogen peroxide (H_2_O_2_), arise as intermediate products during oxygen metabolism. These ROS are extremely reactive radicals that possess the ability to cause protein carboxylation or protein denaturation. An abnormal increase in ROS concentration leads to a disruption of redox equilibrium within the body, resulting in the breakdown of the structure and function of vital cellular macromolecules, including proteins, lipids, and nucleic acids due to oxidative stress. Conditions like myocardial infarction, inflammation, aging, and cancer are all connected to the accumulation of ROS within cells and the reduction of antioxidant enzyme levels. Nanozymes primarily employ peroxidase activity and superoxide dismutase activity to modulate ROS levels, significantly contributing to cellular protection [85].

Ma and coworkers reported iron oxide NPs as carriers for enhanced tumor-targeted delivery of kinetically inert cisplatin(IV) prodrug, but upon activation by intracellular bio-reductive agents, such as glutathione, it causes potent oxidative damage in the tumor cells (Fig. 4D). Cytotoxic version of cisplatin [Pt (II)], leads to the excess generation of superoxide anions (O_2_−). which then gets scavenged by the endogenous superoxide dismutase into H_2_O_2_. Meanwhile, hydrolysis of the iron oxide nanocarrier in the acidic conditions of tumor intracellular environment releases Fe^2+^/Fe^3+^ ions to catalyze the conversion of cisplatin-induced H_2_O_2_ into highly toxic hydroxyl radicals via the Fenton reaction (Fig. 4D). The combination of cisplatin (IV) prodrug and self-immolating iron oxide nanoparticles was beneficial in enhancing the antitumor efficacy with minimal cytotoxicity to healthy cells [77].

## Ultrasound responsive nanozymes

“Ultrasound-responsive nanozymes” refers to a class of NPs that exhibit enzyme-like activity and can be regulated by acoustic waves. These nanozymes are designed to have specific catalytic properties, like natural enzymes, but with the added benefit of being responsive to ultrasound stimulation. The application of ultrasound can trigger changes in the nanozyme structure or function, enabling precise control over their activity. These nanozymes have shown promising potential in various biomedical applications, such as targeted drug delivery, imaging, and therapeutic treatments. The ability to remotely control their catalytic activity using non-invasive ultrasound waves makes them a promising tool in the field of nanomedicine.

Nanosystems having two redox pairs (i.e., Fe^2+^/Fe^3+^ and Cu^+^/Cu^2+^) can be efficient in·OH generation through Fenton or similar reactions under external stimuli. controlled chemotherapeutic drug release and SDT are successful applications of ultrasound specifically in cancer treatments. US creates concentrated shock waves to generate cavitation bubbles, followed by intense local vibration and this acoustic cavitation mechanism was exploited for increased diffusion of the nanozymes, which in turn enhances the Fenton and Fenton-like reaction rates significantly. Therefore, with a suitable integration of photothermally and US-enhanced CDT onto a nanozyme can competently inhibit the tumor progression and recurrence [86]. There are several formulations of nanozymes studied and are under continuous exploration that has varied behaviors with US irradiation and provide efficient bioactivity.

### Nanobubbles

**Fig. 5:**
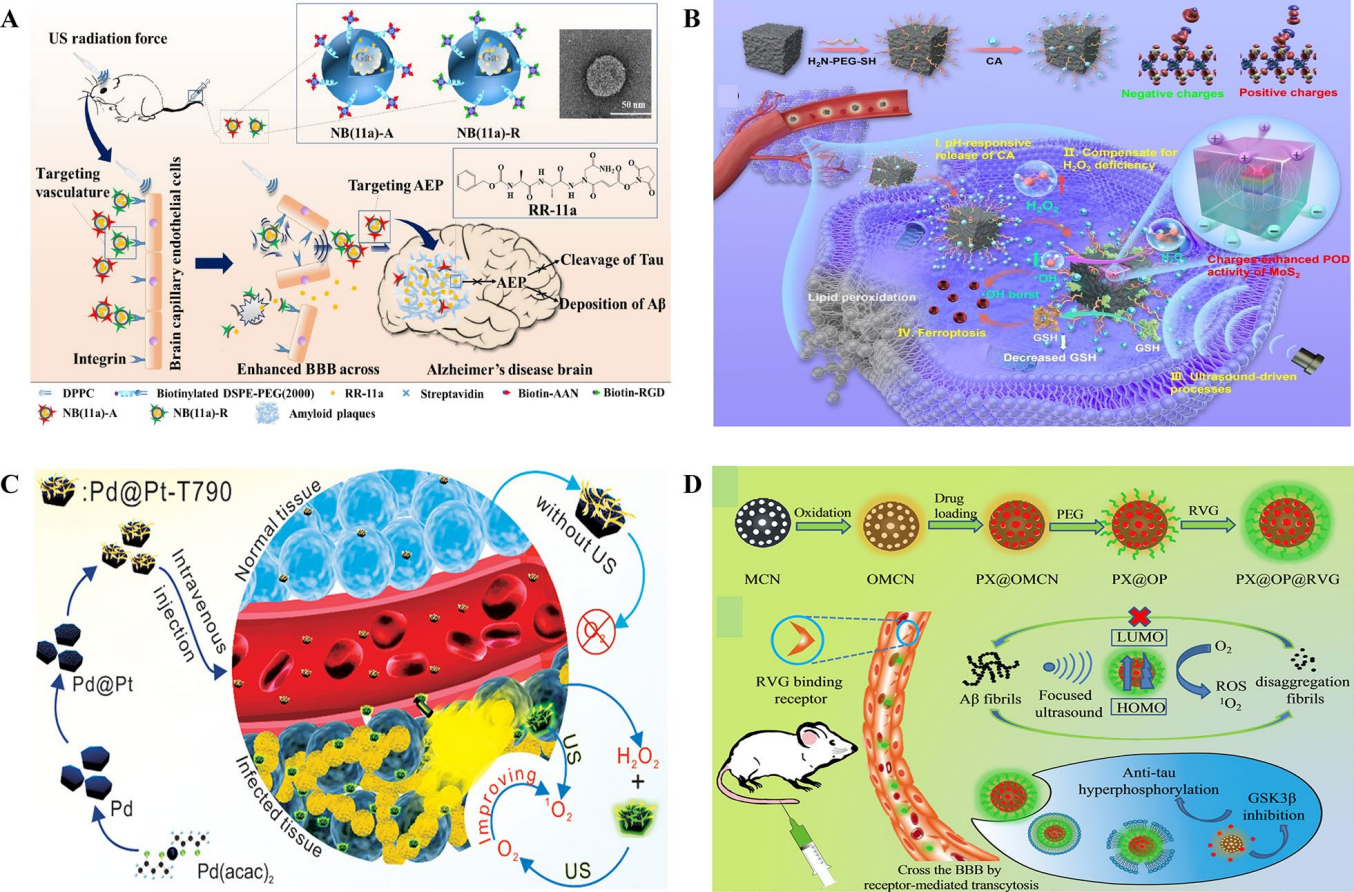
**A** Schematic illustration of the designed RR-11a-loaded and AAN- or RGD-modified nanobubbles for cooperative drug delivery and AEP-targeted AD treatment. NB (11a)-R adhere to endothelial cells and transiently open the BBB upon focused ultrasound oscillations, allowing the rest NBs/localized released RR-11a molecules to enter the brain, and then NB (11a)-A selectively bind with the impaired neurons and deposit therapeutic molecules at the AD lesion. The accumulated RR-11a molecules concurrently inhibit cleavage of Tau and deposition of Aβ in the brain of AD mouse. Reproduced with permission copyright 2022, Elesevier [87]. **B** Schematic illustration of positive and negative charge enhancement of MoS_2_ POD catalytic activity for efficient antitumor therapy. Schematic illustration of the synthesis of BTO/MoS_2_@CA and positive and negative charge promoted dissociation of H_2_O_2_. Piezoelectric catalytic therapeutic process of BTO/MoS_2_@CA under US irradiation. Reproduced with permission copyright 2023, Wiley [88]. **C** Schematic of the US-switchable enzymatic activity of the Pd@Pt-T790 nanoplatform in different tissues. Reproduced with permission copyright 2020, American Chemical Society [89]. **D** Schematic Illustration of the Fabrication of the PX@OP@RVG Nanosystem; NPs cross the BBB by receptor-mediated transcytosis and double-targeted treating process *In-vitro* and *In-vivo*. Reproduced with permission copyright 2018, American Chemical Society [90]

Mi and his colleagues introduced a novel approach to treat AD by targeting asparagine endopeptidase (AEP) with an inhibitor known as RR-11a (Fig. 5A) [87]. The conventional methods of delivering AEP inhibitors directly to the brain have faced significant challenges. In their research, scientists developed ultrasound-responsive nanobubbles (NBs) loaded with RR-11a and modified their surfaces using AAN or RGD peptides to improve the effectiveness of AEP-targeted treatment for AD. These NBs were characterized by their small size, high drug-loading efficiency, and the ability to interact with focused ultrasound and temporarily opening the BBB for facilitating the entry of RR-11a into the brain. Upon BBB opening, NBs with AAN peptides specifically adhered to impaired neurons, leading to the deposition of RR-11a at AD lesions. This combined approach significantly elevated the accumulation of RR-11a in the brain, resulting in the reduction of tau cleavage and the deposition of amyloid plaques in the hippocampus. Cognitive function of AD model mice showed remarkable improvement, approaching normal range observed in control mice without AD. This innovative AEP-targeted nanobubble strategy offers an efficient method for delivering drugs within the brain and enhances the effectiveness of AD treatment. Furthermore, it holds promise for potential applications in other age-dependent neurodegenerative diseases.

### Micelles

Yuanru Zhao et al. introduced a strategy involving prodrugs, namely 3,3’-polyethylene glycol dipropionate-paclitaxel (DP) and 3,3’-polyethylene glycol dipropionate-Ce6 (DC), loaded onto platinum nanozymes (DC@Pt). These prodrugs formed stable micelles, which were further coated with homologous tumor cell membrane to create redox-responsive micelles, termed DPC@Pt@M. This innovative approach was devised for the combined sonodynamic and chemotherapy treatment of colon cancer. The incorporation of PEG-modified sonosensitizer Ce6 and chemotherapy drug PTX (DC and DP) enhanced their water solubility and bioavailability. The presence of -SeSe- bonds in the micelles imparted redox responsiveness, triggered by elevated levels of GSH and H_2_O_2_ within the tumor microenvironment. The platinum nanozymes, acting as catalysts with CAT-like and POD-like properties, facilitated the breakdown of H_2_O_2_ into O_2_ and OH radicals, respectively. This contributed to alleviating hypoxia and enhancing SDT. Additionally, the platinum nanozymes exhibited SOD-like activity, replenishing the consumed H_2_O_2_. The DPC@Pt@M micelles displayed uniformity with the tumor cell membrane, displaying notable biocompatibility and effective targeting both *in vitro* and *in vivo*. Following treatment with US and a combination of SDT and chemotherapy, these novel formulations effectively increased ROS production and efficiently induced apoptosis, leading to improved colon cancer therapy [91].

### Polymer

Wei-Peng Li et al reported a promising polysome, in which H_2_O_2_ was encapsulated in the core with Fe_3_O_4_ which was packed in a shell of polymer forming a microstructure termed as polysome. The formulation is used as a micro US based theranostic agent for conducting imaging and therapy process simultaneously. As an alternative to gaseous or low-boiling-point perfluorocarbons, cost-effective H_2_O_2_ loaded polysome was designed to display US contrast enhancement with better echogenic reflectivity and concurrently employed in ROS-mediated cancer therapy triggered by the micro-US diagnostic system accompanied by MR imaging. The incorporation of H_2_O_2_ plays a pivotal role within the polymeric composition, serving two essential functions: it supplies O_2_ to enhance echogenic reflectivity and generates OH as a therapeutic ROS. The responsiveness of H_2_O_2_/Fe_3_O_4_–PLGA to acidic conditions signifies its potential as a pH-sensitive carrier. This feature becomes particularly advantageous against malignant tumors upon exposure to external US stimulation. The stable polysomes gradually degrade, thereby releasing OH over a period of time. The disruption of polysomes, leading to OH production via the Fenton reaction, is effectively initiated by a micro-US diagnostic system through a nonthermal process. This innovative approach introduces a sensitization-free SDT, showing promise in potentially restraining tumor growth within a mouse model [92].

### Hydrogels

Wang et al. reported the simultaneous encapsulation of sonosensitizer chlorin e6 (Ce6) and Prussian blue based catalytic nanoparticles (PB) into a low melting agarose hydrogel to prepare a multifunctional nano system (PB+Ce6@Hy) for enhanced SDT/PTT. Locally administered PB+Ce6@Hy nanozymes when irradiated by 808 nm laser, it absorbs light energy and converts it into heat energy, causing the hydrogel to respond to the photothermal effect and aids in the on-demand release of Ce6 and PB. Conversely, PB exhibits a continuous catalytic response when interacting with endogenous H_2_O_2_, resulting in the on-site generation of O_2_. This action effectively mitigates the hypoxic microenvironment, subsequently enhancing the efficacy of the successive Ce6-mediated SDT. This synergistic interplay effectively addresses the hurdles associated with SDT, leading to substantial suppression of subcutaneous tumors with minimal associated toxicities. The integration of photothermal therapy (PTT) with an enhanced SDT approach introduces a new perspective to Ce6-driven tumor treatment [71, 93].

Longwei Wang et al reported positive and negative charges on the surface of piezoelectric materials under US, can enhance the POD-like activity of MoS_2_. This can be theoretically explained by the reduced binding energy between MoS_2_ and H_2_O_2_, as well as the promoted dissociation of H_2_O_2_. nano catalyst fabricated with few-layer of MoS_2_ nanosheets on the surface of piezoelectric T-BTO NPs showed multifunctional property, and further linking with pH responsive Polyethylene glycol (PEG)-modified cinnamaldehyde (CA) showed enhanced performance (Fig. 5B). CA formulated with the nanozyme has successfully overcame the shortcomings of free CA molecules such as poor stability, low biological half-life, and systemic toxicity. CA released from the formulation due to the acidic environment in TME served as the initiating catalyst for the production of abundant H_2_O_2_, thereby enhancing tumor selectivity and reducing side effects. Subsequently, the generated H_2_O_2_ undergoes POD like catalytic reaction carried out by BTO/MoS_2_ to liberate highly toxic ^*^OH radicals under US. The nanozyme material was also reported for its capacity to reduce glutathione (GSH) via sulfhydryl group binding, which results in enhanced oxidative stress and reduced expression of peroxidase 4 (GPX4). This further induces the disturbance in redox homeostasis and tumor cell ferroptosis. The pH-responsive CA release-mediated H_2_O_2_ self supply, positive and negative charge enhancement, collective enzymatic activity, makes the BTO/MoS_2_@CA nanozyme, an excellent selective and multifunctiuonal tumor therapy for future application [88].

### Nanoparticles

Duo Sun have reported a nano platform based on Pd@Pt-T790 and used it for catalysis-enhanced SDT to treat MRSA-infected myositis. The well-designed Pd@Pt-T790 nanoparticles exhibits the many advantages over other counterparts like appreciable stability in biological systems which helps in avoiding premature release of the active therapeutic drug, effective accumulation even at the deep-seated bacterial infection as a result of EPR effect, regulation of nanozyme activity by sonosensitizer T790, whereas the US irradiation could effectively activate the catalase-like activity of Pd@Pt-T790, reduced toxicity of nanozyme on normal organs, facilitates abundant oxygen sources for SDT-induced ROS production to significantly enhance the therapeutic efficacy (Fig. 5C). Further, nano systems aids in multimodal imaging for early and accurate diagnosis, helps in real-time monitoring of the treatment or surgical processes. These benefits establish the Pd@Pt-T790 nanotherapeutic platform as a superior approach for treating bacterial infections both *in vitro* and in vivo using US irradiation. This study not only enhances the effectiveness of SDT through US-controllable catalytic oxygen generation, but also offers a potent therapeutic strategy to address deep-seated bacterial infections via US irradiation. Importantly, this method overcomes any limitations associated with tissue penetration [89].Xu et al. emphasize AD as a significant global issue with no effective treatments. AD is characterized by Aβ plaques and neurofibrillary tangles in the brain. The study explores the interactions and synergistic effects between these hallmarks and suggests that targeting multiple aspects of AD may be more promising than focusing on a single target. The research introduces a novel AD multifunctional nanodrug, PX@OMCN@PEG(OP)@RVGs, which efficiently inhibits tau phosphorylation and prevents Aβ aggregation (Fig. 5D). These approaches were shown to significantly improve the cognitive function of transgenic mice with AD. Moreover, the nanodrug was engineered to address the critical challenge of safely crossing the BBB, through modification with the RVG peptide. The study also leverages the photothermal properties of the nanoparticles to enhance BBB permeability, making it possible for the nanodrug to effectively penetrate brain tissue under near-infrared irradiation. This multifunctional nanomedicine, PX@OP@RVG, demonstrated a dual-target treatment capability for AD and showed promise in potentially treating the disease effectively. The study successfully developed a nanosystem triggered by focused ultrasound (PX@OP@RVG) that inhibits Aβ aggregation and reduces cytotoxicity. This effect is achieved through various mechanisms, including PX ultrasonic oxidation, the reduction of phosphorylated tau, and improved BBB penetration. These findings present a promising avenue for the development of nanomedicines that can efficiently target and treat AD. However, further research is necessary before considering clinical applications 90,94.

## Advantages of ultrasound in nanocatalysis

Sonochemistry involves use the energy of ultrasound within the frequency range of 20 kHz to 2 MHz (in contrast to diagnostic US commonly used in medical imaging, which operates within the range of 2 MHz to 200 MHz). This technique employs US to induce chemical, thermal, and physical effects in a solution. The impacts of US encompass several phenomena, including the formation, enlargement, and implosion of gaseous microbubbles in a liquid phase. These effects give rise to intense local heating reaching temperatures of up to 5000 K, exceedingly high pressures nearing 1000 bar, divergent shock waves, acoustic microcurrents, and rapid liquid microjets reaching speeds of up to 100 ms^−1^ [94]. The potential and applications of sonochemistry are closely tied to the selection of specific sonochemical parameters. For instance, the frequency serves as a pivotal parameter, and when dealing with water, lower frequencies (ranging from 20 kHz to 80 kHz) are commonly favored to primarily explore physical effects like microconvection, shockwaves, and microjets. Conversely, higher frequencies (ranging from 150 kHz to 2000 kHz) are more conducive to studying the chemical effects of US, particularly emphasizing the production of hydroxyl radical species (HO·). Conditions induced in a medium subjected to intense US yield a myriad of physicochemical consequences, including shifts in reaction mechanisms and kinetics, emulsification outcomes, erosion, crystallization, precipitation, and more. US is a mechanically favorable wave with notable applications in biomedical domains. Its significance stems from the cavitation effect, sonoluminescence, sonoporation, pyrolysis, and a host of other biophysical and chemical effects. A diverse range of substances have been found to be responsive to the stimulation of ultrasound. This technique boasts safety, noninvasive real-time visualization, painless execution for patients, and easy accessibility. Consequently, US has found extensive use in the realm of disease diagnosis [95]. In addition to its diagnostic applications, focused ultrasound (FUS) is frequently utilized to break down nanocarriers to facilitate drug release or to thermally destroy cancerous tumors via hyperthermia. Consequently, the use of focused US demands careful consideration to prevent inadvertent harm to the neighboring healthy tissues surrounding the intended target area. The utilization of a US diagnostic system offers the advantageous qualities of being innocuous to healthy tissues and providing patients with a pain-free treatment experience [96].

US has become an inevitable tool for a wide range of academia and industrial research fields starting from electrochemistry, food technology, chemical synthesis, materials extraction, nanotechnology, phase separation, surface cleaning, water and sewage treatment, biomedicine, therapy, surgery and etc. Essentially there are three “strands” in ultrasonics research, Sonochemistry originating from chemistry and physics, which is mainly involved synthesis and catalysis aided by cavitation and is an integral part of academic research that. Engineering and processing applications make use of powerful US, primarily in areas such as cleaning, welding, and materials processing within the industrial sector. Meanwhile, diagnostic US, which encompasses non-destructive testing (NDT) and medical scanning, garners significant attention from both academic and industrial circles.

## Neuromodulation and role of ultrasound

Neuromodulation is the therapeutic alteration of neuronal activity in response to physical, chemical, or biological stimuli at the site of interest [97]. It has gained significant attention for its potential to treat various neurological and psychiatric disorders by directly influencing the function of nervous system. Ability of neuromodulation methods to provide precision and specificity gives them an edge over the conventional traditional pharmacological interventions. Mechanisms of neuromodulation depends on the methods employed like electrical stimulation that alter the neuronal conduction and influences overall activity of the nerves [98]. While neuromodulation carried out with drugs and other biomolecules that affect the levels of neurotransmitters and in turn influence the neuronal crosstalk, are termed as chemical or biological neuromodulation respectively [99]. The major advantage observed by experts with respect to neuromodulation is its precision in targeted areas with lesser side effects. Neuromodulation is successful in many of the chronic neurological conditions like neurodegenerative disorders (AD, PD), pain, epilepsy and psychological disorders [100]. The section elaborates on the various methodologies involved in neuromodulation and how ultrasound based neuromodulation is better compared to other physical and chemical methods [101].

## Different methods in neuromodulation

### Chemical methods in neuromodulation

Chemical neuromodulation utilizes chemical agents to modify neural circuit activity, offering a versatile and targeted approach to influence the nervous system at molecular and cellular levels [102]. Key aspects include manipulating neurotransmitter levels, employing pharmacological agents like serotonin reuptake inhibitors (SSRIs), and selectively targeting receptors on neurons, using agonists or antagonists. Optogenetics and chemogenetics, also forms of chemical neuromodulation that often utilize genetic and optical methods,. These methods control neuronal activity with light or specific chemicals [103]. Modulating neurotransmitter precursors or metabolic pathways, such as with L-DOPA, impacts neurotransmitter levels. Chemical neuromodulation is applied in managing various neurological and psychiatric disorders, like using antipsychotic medications for schizophrenia and etc. Individualized treatment is possible through tailored dosages, drug combinations, and durations. Chemical neuromodulation serves scientific research and therapeutic interventions by contributing to advancements in the clinical care for neurological conditions [104]. In summary, chemical methods offer precise means to influence neural circuitry, playing a crucial role in research and therapeutic strategies for neurological and psychiatric conditions.

**Fig. 6:**
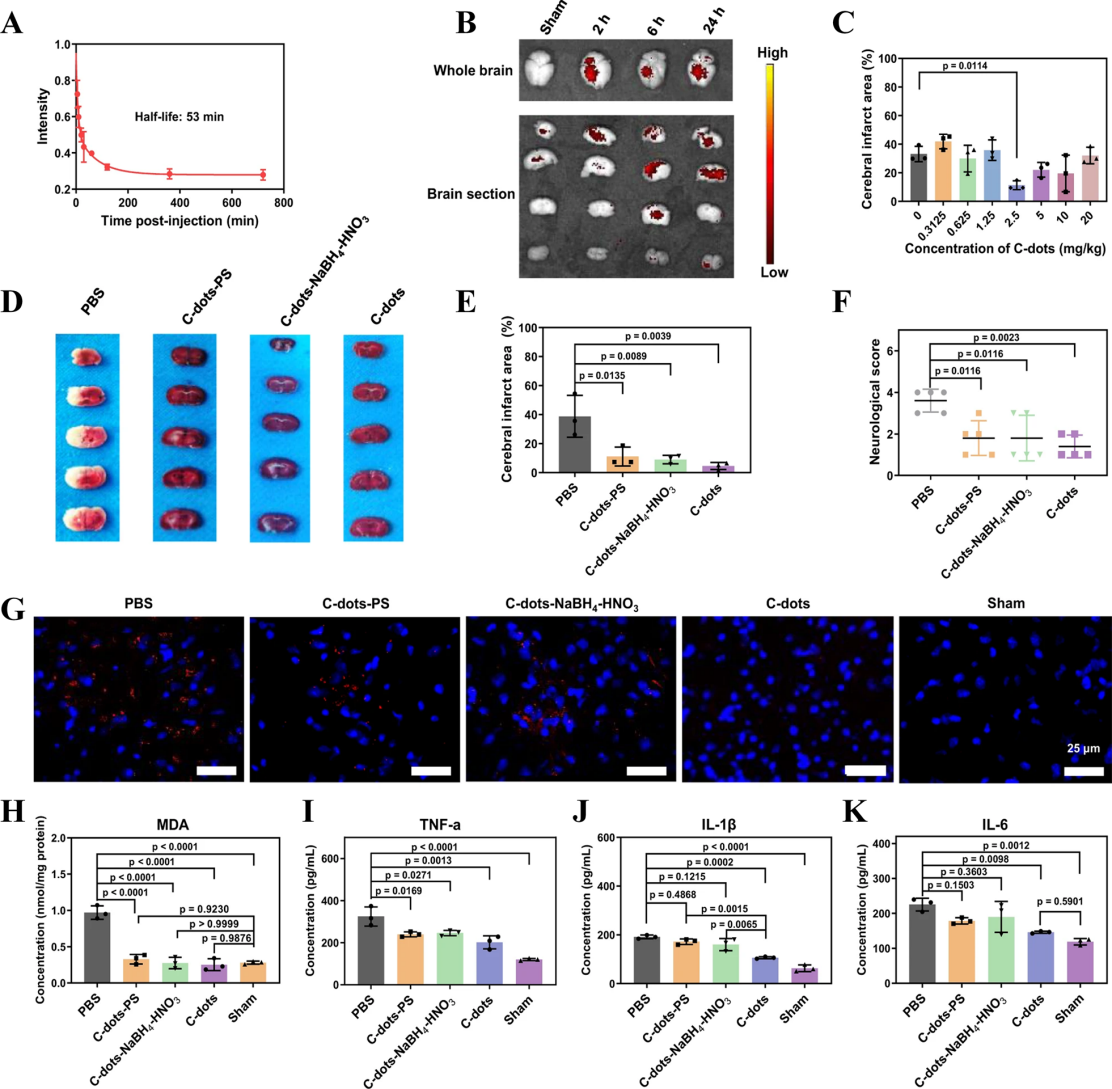
**A** Half-life analysis of C-dot SOD nanozymes in plasma (n = 3 mice). **B** *ex vivo* fluorescence imaging analyses of the accumulations of C-dot SOD nanozymes (labeled with Cy5.5) in the brains of sham (24 h post-injection) and MCAO mice (2, 6, and 24 h post-injection), and corresponding brain sections (n = 3 mice). **C** The cerebral infarcted area analyses of MCAO mice treated with different dosages of C-dot SOD nanozymes for 24 h (n = 3 mice). **D** Representative 2,3,5- triphenyltetrazolium chloride-stained brain sections and **E** quantification of cerebral infarct areas of MCAO mice treated with different C-dot nanozymes (n = 3 mice). **F** Neurological score analyses of the MCAO mice treated with different C-dot nanozymes for 24 h (n = 5 mice). **G** Representative images of TUNEL staining in the brain sections (n = 3 mice, scale bar = 25 μm), **H** Malondialdehyde (MDA) assay in the brain homogenate, and ELISA assay of inflammatory factors (**I**) TNF-α, **J** IL-1β and (K) IL-6 of the infarcted brain of MCAO mice treated by different C-dot nanozymes (n = 3 mice). P values are determined with one-way ANOVA Tukey’s multiple comparisons test. Reproduced with permission copyright 2023, Nature communication [24]

Gao and co workers reported carbon dot (C-dot) nanozymes exhibiting SOD-like activity, effectively scavenging superoxide anions, a major source of reactive oxygen species (Fig. 6) [24]. These nanozymes demonstrate catalytic activity exceeding 10,000 U/mg, compared to natural enzymes. By implementing specific chemical modification and theoretical computations, it is demonstrated that the SOD-like capability of C-dots is contingent on the interaction of hydroxyl and carboxyl groups with superoxide anions. Furthermore, electron transfer is facilitated by the carbonyl groups, which are conjugated with the π-system.The mechanism involves hydroxyl and carboxyl groups binding superoxide anions, and carbonyl groups facilitating electron transfer. C-dot based SOD nanozymes, with intrinsic targeting ability, protect neuron cells in an ischemic stroke model, by enhanced accumulation preferentially in the damaged brain regions. The nanozymes effectively alleviate neurological damage post-stroke, reducing infarcted areas, apoptotic cells, and lipid peroxidation. They also lower proinflammatory cytokines, showcasing potential for treating oxidative stress-related diseases. The study establishes optimal dosage and therapeutic effects, highlighting the nanozymes’ promise for treating ischemic stroke. Furthermore, C-dot nanozymes exhibit biocompatibility, cross cell membranes efficiently, and offer advantages over natural enzymes, making them a promising substitute with broad application potential in medical, and biological fields.

### Physical methods in neuromodulation

Physical methods in neuromodulation utilize external stimuli such as electrical, magnetic, thermal, and mechanical forces to modify neural activity, offering less invasive alternatives to traditional interventions [105]. Electrical techniques, like transcranial direct current stimulation (tDCS) and transcranial alternating aurrent stimulation (tACS), are employed for conditions like depression, chronic pain, and cognitive enhancement. Magnetic Stimulation, notably transcranial magnetic stimulation (TMS), induces brain currents to treat depression, schizophrenia, and chronic pain [106]. Ultrasound neuromodulation, specifically low-intensity focused ultrasound (LIFU), and optical stimulation via optogenetics, target neural activity with ultrasound and light. Thermal neuromodulation, which utilizes temperature changes, is applied in epilepsy and chronic pain treatments. Invasive strategies include vagus nerve stimulation (VNS) and deep brain stimulation (DBS), involving electrical stimulation of the vagus nerve and brain regions, respectively, were applied for conditions like epilepsy, depression, PD, and essential tremor [107]. Mechanical vibration and electric field stimulation are emerging methods explored for pain management, rehabilitation, and cognitive function improvement [108]. These varied approaches underscore the active research and potential for novel treatments in neurological and psychiatric conditions.

A recent investigation on stimulation of neurons through focused ultrasound involves the opening of calcium-selective ion channels activated by mechanical forces acting on the neural membrane [109]. The activation of mechanosensitive ion channels initiates a calcium influx, leading to the activation of nonselective TRPM4 channels and subsequent sodium influx. Consequently, membrane depolarization triggers T-type calcium channels, causing further calcium influx. The activation of mechanosensitive channels and the resulting calcium influx is then enhanced through the indirect activation of other ion channels, collectively resulting in stimulation. Importantly, this study demonstrated that previously suggested mechanisms, such as temperature changes, cavitation formation, and membrane deformation, do not significantly impact the stimulation process.

### Biological methods in neuromodulation

Biological methods in neuromodulation harness the body's own biological processes or introduce biological agents to influence neural activity, targeting molecular and cellular pathways. Key strategies include neurotransmitter modulation through pharmacotherapy, where drugs alter neurotransmitter levels or receptor activity, commonly used for mood disorders [110]. Neurotrophic factors, particularly brain-derived neurotrophic factor (BDNF), promote neuron growth and are explored for neurodegenerative diseases and cognitive enhancement. Cytokine modulation via anti-inflammatory agents targets neuroinflammation, while gene therapy involves gene delivery to cells, often combined with techniques like optogenetics. Stem cell therapy, aiming to replace damaged neurons, is researched for conditions like PD [111]. Immunomodulation, such as immunotherapy, addresses immune responses in disorders like multiple sclerosis. Endogenous modulation enhances natural opioids like endorphins for pain management. Hormonal modulation via hormone therapy impacts neural activity, with applications like estrogen replacement in postmenopausal women for mood regulation. Microbiota-Gut-Brain axis modulation investigates the impact of gut-brain communication, using probiotics and prebiotics [112]. Lastly, exosome therapy utilizes vesicles containing biomolecules for targeted therapeutic delivery to the brain. These diverse biological methods range from traditional pharmacotherapy to innovative approaches like stem cell and exosome therapy, offering new avenues for treating neurological and psychiatric conditions.

**Fig. 7:**
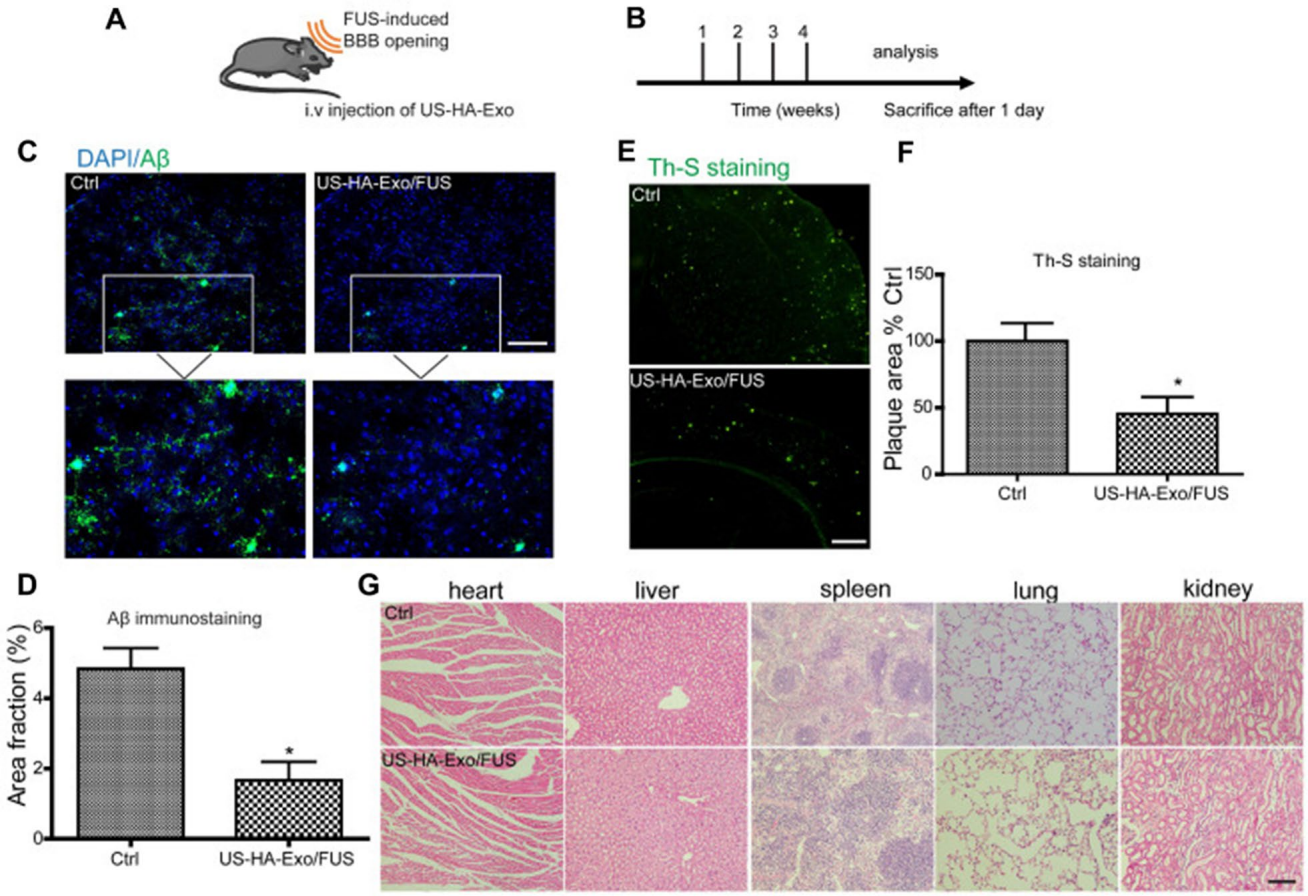
Brain-targeted delivery of US-HA-Exo combined with FUS-mediated BBB opening. **A**, **B** Schematic of FUS-BBB opening-assisted delivery of exosomes. **C** Brain sections obtained from 10-month-old APP/PS1 mice were immuno-stained for Aβ, scale bar: 100 µm. **D** The percentage area of positive amyloid-β stainning was quantified. **E**, **F** Aβ plaques in the brain were detected by thioflavin-S staining (scale bar: 100 µm), and quantified. **G** H&E staining of major organs of mice, scale bar: 100 µm [113]. Reproduced with permission copyright 2021, Ivyspring International Publisher

Deng et al investigates the use of ultrasound to enhance the release of exosomes from astrocytes, aiming to alleviate neurotoxicity induced by amyloid-β (Fig. 7) [113]. The study demonstrates that focused ultrasound stimulation increases the secretion of exosomes from astrocytes. These augmented exosomes, in turn, exhibit a neuroprotective effect by mitigating amyloid-β-induced neurotoxicity. The findings suggest a potential therapeutic approach for AD, leveraging ultrasound to enhance the beneficial effects of exosomes in neuroprotection. This non-invasive method holds promise in developing novel strategies to counteract the detrimental effects of amyloid-β and improve neuronal health in the context of neurodegenerative diseases. Further research is warranted to explore the full therapeutic potential and mechanisms underlying ultrasound-mediated exosome release from astrocytes in the treatment of Alzheimer's and related disorders.

### Ultrasound based neuromodulation

Ultrasound-based neuromodulation, utilizing low-intensity ultrasound waves in a non-invasive manner to influence neural activity, is increasingly recognized in both neuroscience and therapeutic fields [114]. Its working principle involves mechanical impacts on neurons, such as inducing vibrations or altering pressure, which in turn modulate ion channels and affect neuronal excitability. This technique stands out for its precise targeting capabilities, enabling focused treatment on specific areas of the brain without disturbing nearby regions. Differing from other methods, ultrasound has the ability to reach deeper brain structures, broadening its range of use [115]. Being non-invasive, it eliminates the risks tied to surgical procedures and implanting electrodes, enhancing safety. In research, ultrasound is used to investigate brain function by stimulating or inhibiting brain areas, aiding in understanding cognitive and motor functions [116]. Therapeutically, it's explored for disorders like PD, epilepsy, and chronic pain, offering a potential alternative to traditional treatments with fewer side effects. While current studies indicate safety within medical imaging parameters, long-term effects and side effects require further research. Challenges include accurate targeting, real-time monitoring, individual response variability, and parameter optimization [117]. Overall, ultrasound-based neuromodulation offers a blend of precision, depth penetration, and non-invasiveness, with significant potential in management of neurological disorder, and has drawn the interest of several researchers to fully understand and optimize the therapeutic strategies.

## Role of nanomedicine and nanozymes in ultrasound based neuromodulation

**Fig. 8:**
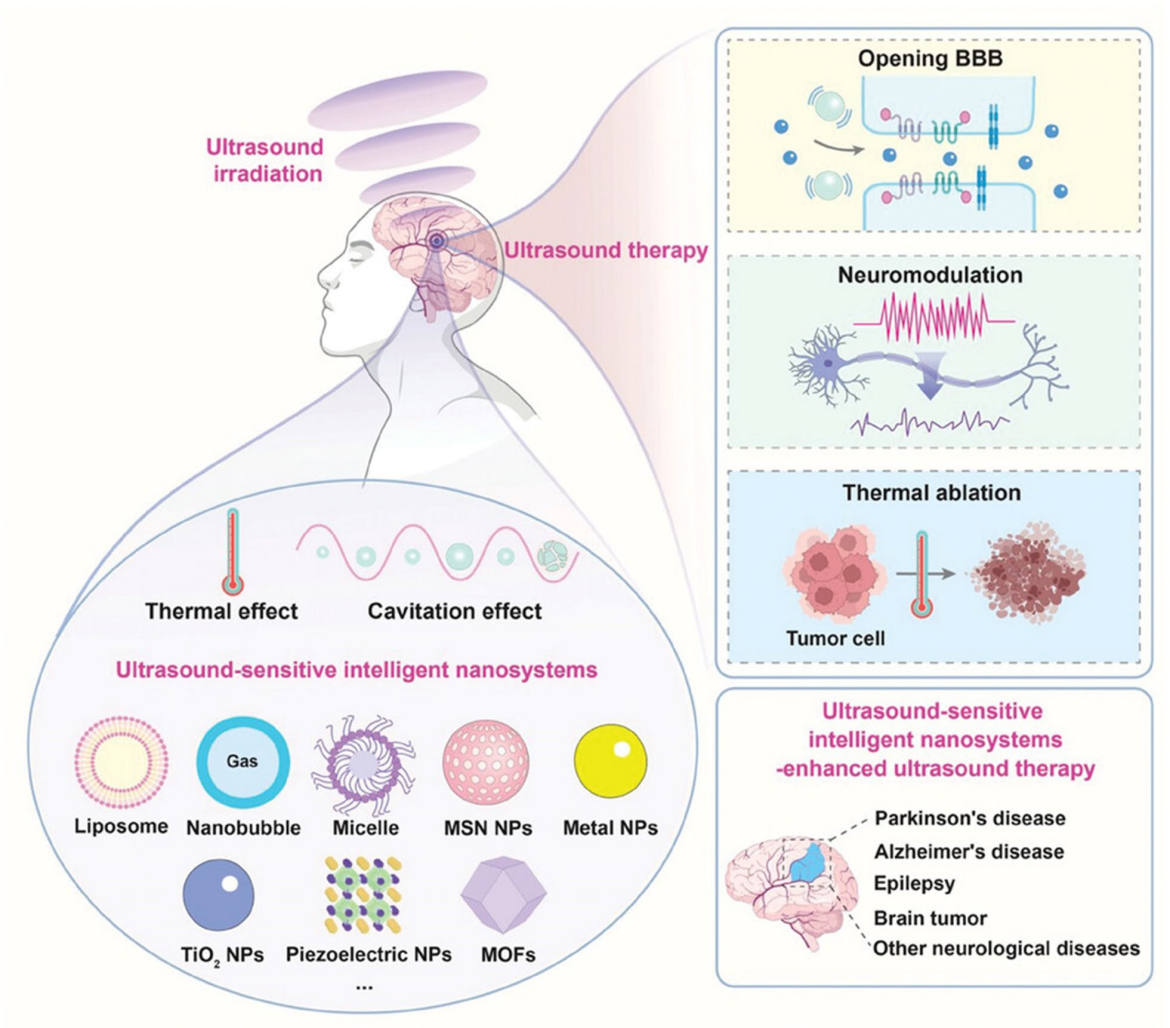
Schematic illustration of USINs-enhanced ultrasound therapy for neurological diseases. MSN, mesoporous silica nanoparticle; MOFs, metal–organic frameworks. [28]. Reproduced with permission copyright 2023, Wiley

Nanomedicine and nanozymes are becoming integral to enhancing ultrasound-based neuromodulation, merging nanotechnology with neuroscience. This approach uses low-intensity ultrasound waves for non-invasive modulation of neural activity. Nanozymes, with their targeted delivery capabilities, allow precise localization and controlled release of therapeutic agents in specific regions of interest [28]. They boost the efficiency of neuromodulation by responding to ultrasound stimulation, which can lead to the release of therapeutic compounds and activation of biological pathways, resulting in improved treatment outcomes. In the realm of bioimaging and monitoring, nanoparticles and nanozymes enhance imaging, enabling real-time observation of neuromodulation effects on the brain [118]. This aids in personalizing treatments and tracking therapeutic progress. Nanozymes, with their catalytic activities, can catalyze reactions beneficial for neural repair and regeneration, complementing ultrasound neuromodulation effects. Nanomedicine can potentially reduce side effects typical of conventional drug therapies by focusing treatment and reducing systemic exposure. The combination of nanomedicine with ultrasound neuromodulation opens new treatment avenues, such as modulating cellular pathways uniquely responsive to ultrasound [119]. Importantly, nanozymes in these applications are designed for biocompatibility and safety, crucial for patient health. Overall, integrating nanomedicine and nanozymes with ultrasound-based neuromodulation represents a significant advancement in neurological treatments, offering more precise, efficient, and tailored therapies (Fig. 8). As research continues, this integration is expected to yield innovative solutions for complex neurological conditions.

## Neuromodulation by ultrasound responsive nanozymes

The integration of ultrasound-responsive nanozymes in neuromodulation combines the catalytic capabilities of nanozymes with the precision of ultrasound for up/downregulated neural activity. Nanozymes, with their enzyme-like activities, respond to ultrasound waves, leading to structural changes and activation of their catalytic functions. We have summarised various nanozymes and their potential application in Table 1. This synergy enables highly localized neuromodulation, enabling to target specific brain region and on-demand release of neuromodulatory substances, potentially enhancing treatment efficacy and offering adaptable, personalized therapy. Ensuring biocompatibility and safety of these nanozymes is an essential step, that is required to reduce cytotoxicity and immunogenic responses. which can be achieved by coatings with biocompatible materials. These nanozymes also hold potential in imaging and monitoring neural activity through detectable signals. Optimization of ultrasound parameters like intensity and frequency enable the fine-tuning of neuromodulatory effects to suit individual patient needs and various neurological conditions. Despite the promise shown by ultrasound responsive, challenges such as optimizing nanozyme design, understanding the molecular interactions between ultrasound and nanozymes, and addressing long-term effects and biocompatibility are still unresolved. In summary, ultrasound-responsive nanozymes in neuromodulation represent a significant advancement in precision medicine for treating neurological disorders, warranting ongoing research to harness their full potential.

**Table 1:** The potential application of nanozymes in neurological disease

Nanozyme material	Disease	Type of functions	Signaling mechanism	Therapeutic effects	Reference
Pd@PEG@Bor	AD	SOD, CAT	ROS↓; inhibit Aβ plaque deposition	Alleviate neuron loss, and neuroinflammation; improve cognitive impairment	[121]
Fe-Nx SANs	AD	POD	Enhance the detection performance of Aβ 1-40	Ultrasensitive colorimetric immunoassay	[132]
PEG-Fe_3_O_4_	AD	SOD, CAT	ROS↓; PECAM-1↑; claudin5↑; ZO-1↑	Improve neuroblast differentiation in the hippocampal dentate gyrus	[133]
KLVFF@Au-CeO_2_	AD	SOD, CAT	ROS↓; inhibit Aβ	Aggregation and degrade Aβ fibril; improve the cognitive function	[134]
TPP-MoS_2_ QDs	AD	SOD, CAT	ROS↓; IL-1β↓; IL-6↓; TNF-α↓; TGF-β↑	Degrade Aβ deposits; attenuate inflammatory	[135]
KD8@N-MCNs	AD	SOD, CAT	ROS↓; IL-1β↓; TNF-α↓	Decrease Aβ deposits, ameliorate memory deficits, and alleviate neuroinflammation	[136]
2D V2C MXenzyme	PD	SOD, CAT, GPx	ROS↓; tyrosine hydroxylase↑; IBA-1↓	Inhibit inflammation	[137]
Lf-Au-Bi2Se3	PD	SOD, CAT, GPx	ROS↓	Improve the memory and mobility, protect mitochondria, suppress dopaminergic neuron loss in the substantia nigra pars compacta	[138]
Mnf	PD	SOD, CAT, GPx	ROS↓	Inhibit caspases-3/7 activation	[139]
PBzyme	PD	SOD, CAT	ROS↓; NLRP3 inflammasomes↓; caspase-1↓; GSDMD↓	Reduces dopaminergic degeneration and inhibits neuroinflammation; alleviates motor deficits, attenuates the damage of mitochondrial membrane potential, and rescues dopaminergic neurons	[140]
Fe_2_NC@Se	Stroke	SOD, CAT, GPx	ROS↓	Improve neurological function, decrease brain infarct volumes and edema, ameliorate brain injury; inhibit ASK1/JNK pathway	[141]
PNzyme/MnO_2_	Stroke	SOD, CAT	ROS↓; TNF-α↓; IL-6↓	Inhibit inflammation; reduces cerebral infarction; improve neurological function	[25]
Pt/CeO_2_	Brain injury	SOD, CAT, GPx	ROS↓; RNS↓	Improve the wound healing of neurotrauma and reduce neuroinflammation	[142]
PtPdMo triM	Brain injury	SOD, CAT	ROS↓; RNS↓	Suppress neuroinflammation; attenuate brain injury and improve memory	[60]
Cr/CeO_2_	Brain injury	SOD, CAT, GPx	ROS↓; RNS↓; MMP-9↓	Improve the wound healing and reduce neuroinflammation; improve neuronal cognition	[143]

## Effect of stimuli responsive nanozymes in AD therapy

Nanozymes with enzyme-like catalytic activities and controllable response based on the external stimuli like pH, temperature etc, has been applied in management of various complex diseases. In this section we have discussed about some potential roles stimuli-responsive nanozymes could play a role in treatment of AD.

### Targeted Drug Delivery

Stimuli-responsive nanozymes can be designed to respond according to specific conditions in the brain associated with AD, such as elevated levels of certain enzymes or changes in pH. This responsiveness can be utilized for targeted drug delivery, releasing therapeutic agents at the site of action.

**Fig. 9:**
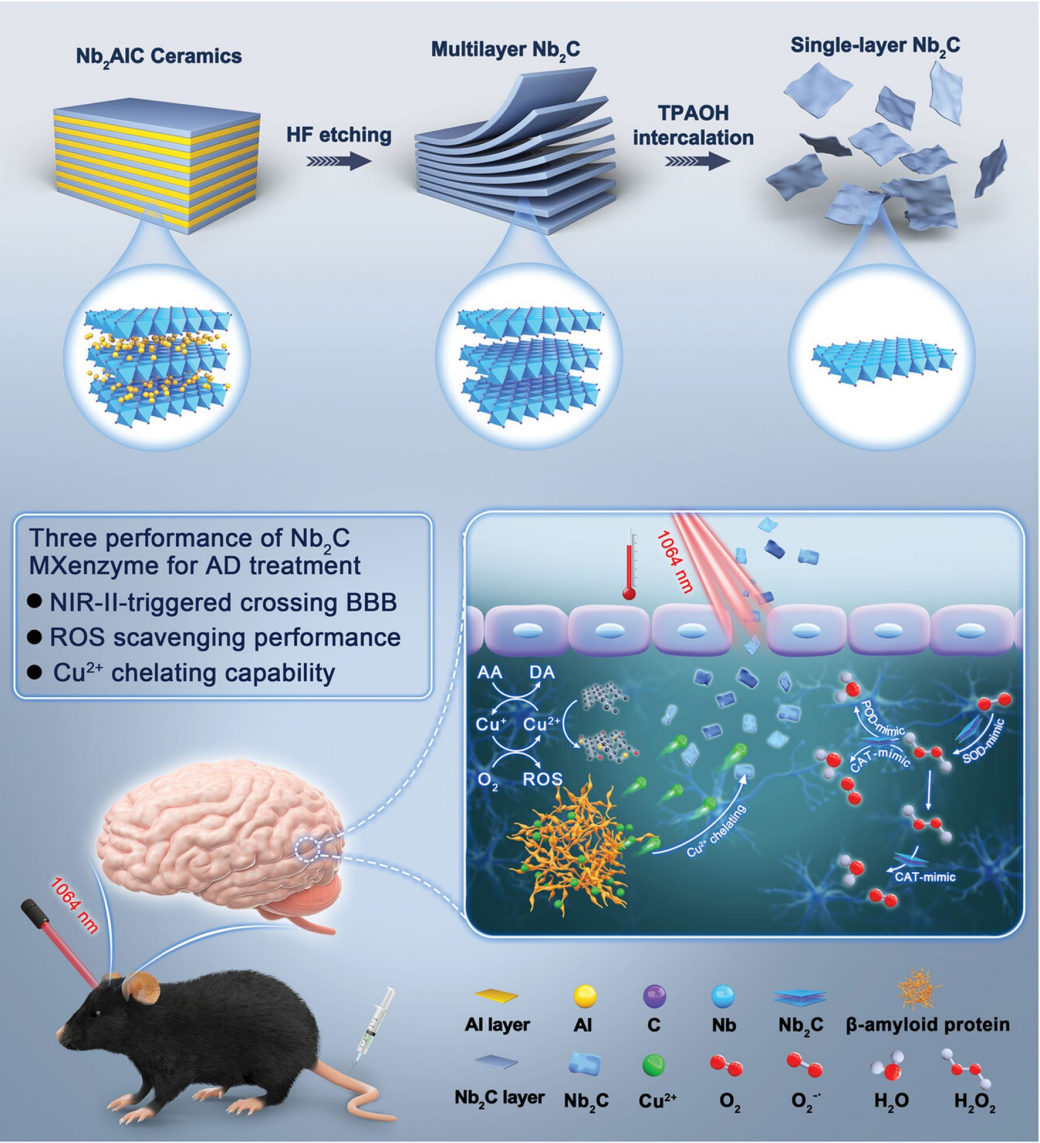
Schematic diagram showing the delamination and disintegration of ultrathin Nb_2_C nanosheets from bulk Nb_2_AlC ceramics, including HF etching and TPAOH intercalation. Schematic diagram of the unique functionality of 2D Nb2C MXenzyme with high BBB permeability under NIR-II irradiation to capture superfluous Cu^2+^ and multiple enzyme-mimicking properties to catalyze ROS scavenging, primarily mimicking SOD-, CAT-, and POD-like activities [120]. Reproduced with permission copyright 2022, Wiley

Du et al reported transition-metal dyshomeostasis, particularly an excess of copper ions (Cu^2+^), has been identified as a critical factor in the pathogenesis of Alzheimer's disease (AD) [120]. Cu^2+^ catalyzes the production ofROS, leading to neuroinflammation and neuronal cell apoptosis. This study focuses on developing a robust chelating agent capable of binding toxic Cu^2+^ and scavenging over-generated ROS, essential for AD treatment (Fig. 9). A 2D niobium carbide (Nb2C) MXene-based nano-chelator (termed MXenzyme) is engineered, demonstrating effectiveness in suppressing Cu^2+^-induced Aβ peptide aggregation and acting as a nanozyme to eliminate excess cellular ROS. The underlying mechanisms are explored through computational simulation. Notably, the Nb2C MXenzyme exhibits a photothermal effect under near-infrared laser irradiation, enhancing BBB permeability, a significant advantage over conventional anti-AD agents. The engineered Nb2C MXenzyme selectively captures Cu^2+^, preventing coordination with Aβ aggregates and protecting neuronal cells from Cu^2+^-related toxicity. It also mimics natural antioxidant enzymes (like SOD and CAT) to alleviate mitochondrial and neuroglial damage by scavenging excess ROS. Theoretical simulations have been employed to elucidate the mechanisms of selective Cu^2+^ chelation and ROS scavenging. In contrast to traditional agents, the Nb2C MXenzyme under near-infrared laser irradiation shows enhanced BBB permeability, supported by *in vitro* and *in vivo* models. The study demonstrates that the Nb2C MXenzyme is biocompatible and easily excreted, highlighting its potential for clinical translation not only in AD treatment but also in other neurodegenerative diseases. This is attributed to its antioxidant enzyme-mimicking properties, effective BBB penetration, and ease of formulation process. The Nb2C MXenzyme represents a promising approach for tackling transition-metal dyshomeostasis and ROS-mediated central nervous system diseases.

### Oxidative Stress Reduction

AD is associated with oxidative stress, and nanozymes with antioxidant properties could help alleviate this stress. Stimuli-responsive nanozymes could be designed to release antioxidants in response to oxidative stress, providing a dynamic and responsive treatment approach.

**Fig. 10:**
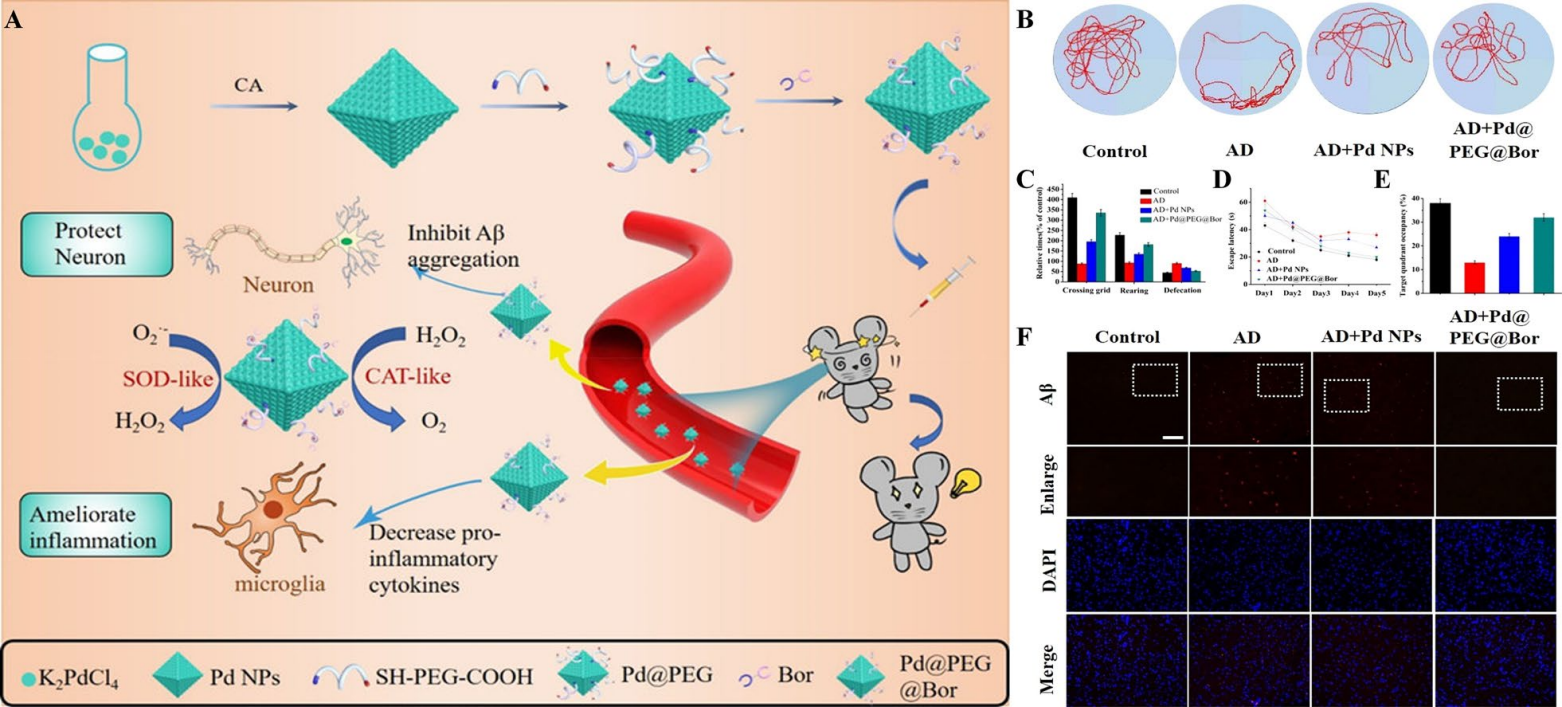
**A** Schematic Chart of Pd@PEG@Bor Synthesis and Schematic Diagram of the Pd@PEG@Bor Mechanism of AD Treatment. Right panel: **B** Amelioration of memory deficits and reduction of Aβ deposits during treatment. Path tracings of different groups in a probe trial. **C** Open-field test involving the numbers of the crossing grid, rearing, and defecation. **D** Latency time of each group in hidden platform learning. **E** Time spent on the target quadrant in a probe trial. **F** Representative images of amyloid plaques stained by the immunofluorescence technique in the cortex region from AD mice (scale bar = 50 μm). Aβ1–42-stained plaques (red); DAPI (blue) [121]. Reproduced with permission copyright 2021, American Chemical Society

Jia et al. reported oxidative stress inAD, often associated with mitochondrial dysfunction, Aβ deposition, and neurofibrillary tangles (NFTs) [121]. Octahedral palladium nanoparticles (Pd NPs) are recognized for their antioxidant enzyme-like properties and biocompatibility but face challenges in penetrating the BBB. To overcome this, a borneol (Bor)-modified octahedral palladium nanozyme platform (Pd@PEG@Bor) was developed to target intracellular ROS and enhance cellular penetrability. Through both *in vitro* and *in vivo* studies, Pd@PEG@Bor has been shown to effectively reduce ROS and Ca^2+^ levels, maintain mitochondrial membrane potential, and protect mitochondria in SH-SY5Y cells (Fig. 10). It accumulates rapidly in the brains of AD mice, reducing symptoms like Aβ plaque deposition, neuron loss, and neuroinflammation, and improves cognitive functions such as learning and memory. Pd@PEG@Bor offers improved BBB penetration and cellular uptake over unmodified Pd NPs without loos of its antioxidant efficacy. It also displays good stability and biocompatibility, scavenging intracellular ROS, maintaining mitochondrial balance, reducing Aβ accumulation, and alleviating neuroinflammation. This research presents a potential new therapeutic strategy for AD treatment, emphasizing the role of Pd@PEG@Bor nanozymes in regulating ROS levels and targeting key pathological features of AD. The findings highlight the potential of nanozymes in AD therapy, suggesting a promising approach for hampering the progression of this neurodegenerative disease.

### Amyloid Beta Degradation

Amyloid beta (Aβ) plaques are a hallmark of AD. Nanozymes could potentially be engineered to degrade or modify Aβ aggregates. The stimuli-responsive nature could allow for targeted action when specific conditions associated with AD are detected.

Li et al. reported a novel therapeutic strategy for AD utilizing a cerium oxide-based system, functioning as a caged metal chelator [122]. This approach combines controlled-release mechanisms with glucose-coated cerium dioxide nanoparticles (G-CeO_2_ NPs) to form a unique dual delivery platform. Specifically designed to respond to H_2_O_2_, this system releases antioxidants and metal chelators in a targeted manner, focusing on the key aspects of AD pathology, including oxidative stress and amyloid-beta peptide aggregation. The platform's H_2_O_2_-responsive nature facilitates controlled drug release, aiming for a more targeted and effective treatment of AD. This approach showcases the potential of integrating nanotechnology with responsive drug delivery systems to address neurodegenerative diseases. The NPs involved in this system are particularly effective in reducing Aβ aggregation, decreasing cellular ROS, and protection against cellular damage linked to AD. Given their excellent biocompatibility, selective targeting of toxic metal ions, ability to cross the blood-brain barrier, and efficient metal chelator release, these nanoparticles are considered a promising option for *In vivo* biomedical applications in AD treatment.

### Diagnostic Imaging

**Fig. 11:**
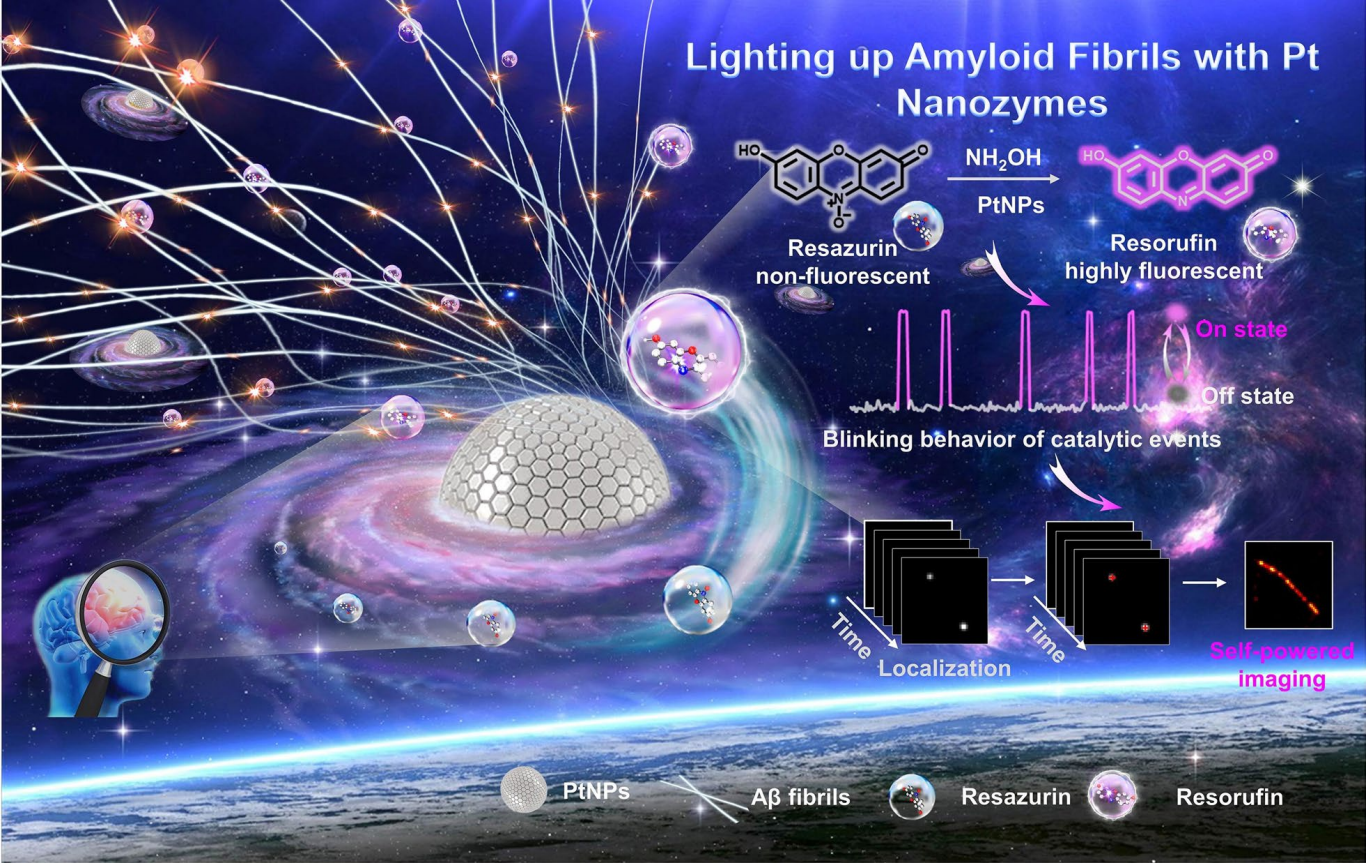
Schematic illustration of the catalytic reaction and imaging method. Pt nanozyme-catalyzed continuous generation of fluorescent tags Rf for the self-powered imaging of Aβ aggregates [123]. Reproduced with permission copyright 2023, Elsevier

Stimuli-responsive nanozymes may also be employed in diagnostic imaging techniques for AD. By responding to specific biomarkers or conditions associated with AD, these nanozymes could enhance the imaging contrast, aiding in early detection and monitoring of the disease. Wang et al. reported on the crucial role of Aβ peptide aggregation in AD and the necessity of visualizing Aβ1-40 aggregates for understanding its pathological mechanisms [123]. Conventional fluorophores, typically used for labeling Aβ aggregates, face challenges like poor photostability and high background noise. To address these issues, the study introduces a self-powered imaging (SPI) strategy that employs PtNPs as nanozymes. These PtNPs catalyze the generation of fluorescent resorufin (Rf), effectively tagging Aβ1-40 aggregates for imaging. This nanozyme-driven SPI strategy enables nanoscopic imaging of Aβ1-40 aggregates through single-molecule localization microscopy and allows visualization of the inhibitory effects of photosensitizers on Aβ1-40 fibrillation with high resolution (Fig. 11). PtNPs facilitate the continuous generation of Rf by catalyzing the deoxygenation of resazurin (Rz), and Rf shows enhanced fluorescence when bound to Aβ1-40 aggregates. This approach is used for microscopic imaging of Aβ1-40 fibrillation and disaggregation both *in vitro* and *in vivo*. The SPI method has the ability to provide nanoscopic imaging of Aβ1-40 aggregates and this is based on modulating the reactant concentration, utilizing the low “on/off” duty cycle and high photon output of the catalytic events. Moreover, the SPI strategy demonstrates a significant inhibitory effect of photosensitizers on Aβ1-40 fibrillation. The versatility of this method extends beyond Aβ1-40 fibril imaging, as the substrate molecules can be modified to detect other biomolecules, and other organic compounds with similar “turn-on” effects can be used. This innovative nanozyme-driven SPI approach offers a general platform for real-time fluorescent imaging of complex biological structures, with significant implications for the study and treatment of AD and other neurodegenerative diseases.

### Antioxidant Therapy

Oxidative stress is implicated in the progression of AD. Stimuli-responsive nanozymes with antioxidant properties can be designed to release antioxidants in response to elevated levels of ROS or other indicators of oxidative stress. This targeted antioxidant therapy can help mitigate oxidative damage in the brain.

**Fig. 12:**
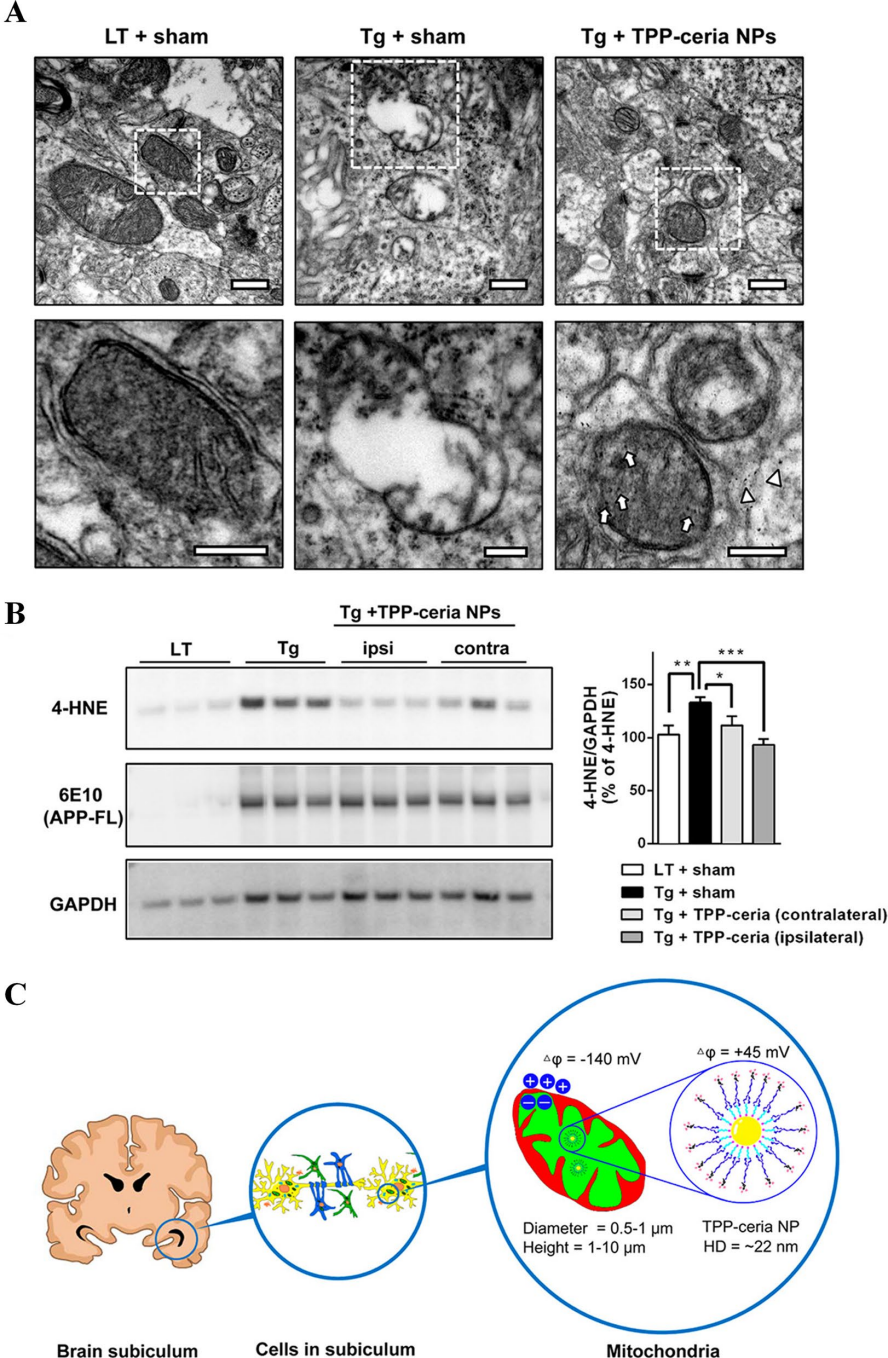
TPP-ceria NPs restore mitochondrial morphology and reduce oxidative stress. **A** TEM images showing representative mitochondrial morphologies of LT + sham, Tg + sham, and Tg + TPP-ceria NPs. Scale bar = 500 nm (n = 4 per group). Magnified images of the boxed areas are shown below. Arrows and arrowheads indicate TPP-ceria NPs in mitochondrial matrix and cytosol, respectively. Scale bar = 250 nm. **B** Western blot analysis for oxidative stress markers in 5XFAD mice treated with TPP-ceria NPs (n = 3 per group). Data were normalized with respect to the signal of GAPDH. Statistical analysis was performed using an ANOVA test. Error bars represent 95% CIs. *p < 0.05; **p < 0.01; ***p < 0.001; LT + sham: littermate mice; Tg + sham: 5XFAD mice. **C** TPP-ceria NPs localize to mitochondria of subicular cells due to their small hydrodynamic diameter (22 nm) and highly positive charge (+45 mV) [124]. Reproduced with permission copyright 2016, American Chemical Society

Kwon et al. reported that mitochondrial oxidative stress, primarily stemming from the abnormal generation of ROS due to mitochondrial dysfunction, plays a vital role in the pathology of neurodegenerative diseases, particularly AD [124]. Ceria (CeO_2_) NPs, recognized for their efficient ROS scavenging ability by alternating between Ce^3+^ and Ce^4+^ oxidation states, have been identified as a potential therapeutic solution. The study focuses on developing triphenylphosphonium-conjugated CeO_2_ NPs, specifically designed to target mitochondria. These NPs effectively localize to the mitochondria and have been shown to suppress neuronal death in a 5XFAD transgenic AD mouse model. They also reduce reactive gliosis and morphological mitochondrial damage observed in these models, emphasizing their therapeutic potential in addressing mitochondrial oxidative stress in AD. The triphenylphosphonium-conjugated ceria NPs (TPP-ceria NPs) are small, positively charged, and demonstrate efficient mitochondrial localization in various cell lines. Their biocompatibility and capacity to effectively scavenge mitochondrial ROS helped to reduce oxidative stress, as proven by *in vitro* and *in vivo* studies. Importantly, the TPP-ceria NPs suppress neuronal death and mitigate reactive gliosis in the AD mouse model, representing a novel approach in mitochondrial therapeutics aimed at counteracting neuroinflammation (Fig. 12). This research marks a significant advancement in the treatment of AD and other neurodegenerative diseases, underscoring the potential of triphenylphosphonium-conjugated ceria NPs in targeted mitochondrial therapy. The development and application of these nanoparticles offer a promising strategy for addressing the challenges of neurodegenerative conditions.

### Responsive Release of Neuroprotective Agents

Nanozymes can be loaded with neuroprotective agents, and their release can be controlled in response to specific stimuli. This approach allows for the timely and targeted delivery of neuroprotective substances, which can help preserve neuronal function and prevent further degeneration.

**Fig. 13:**
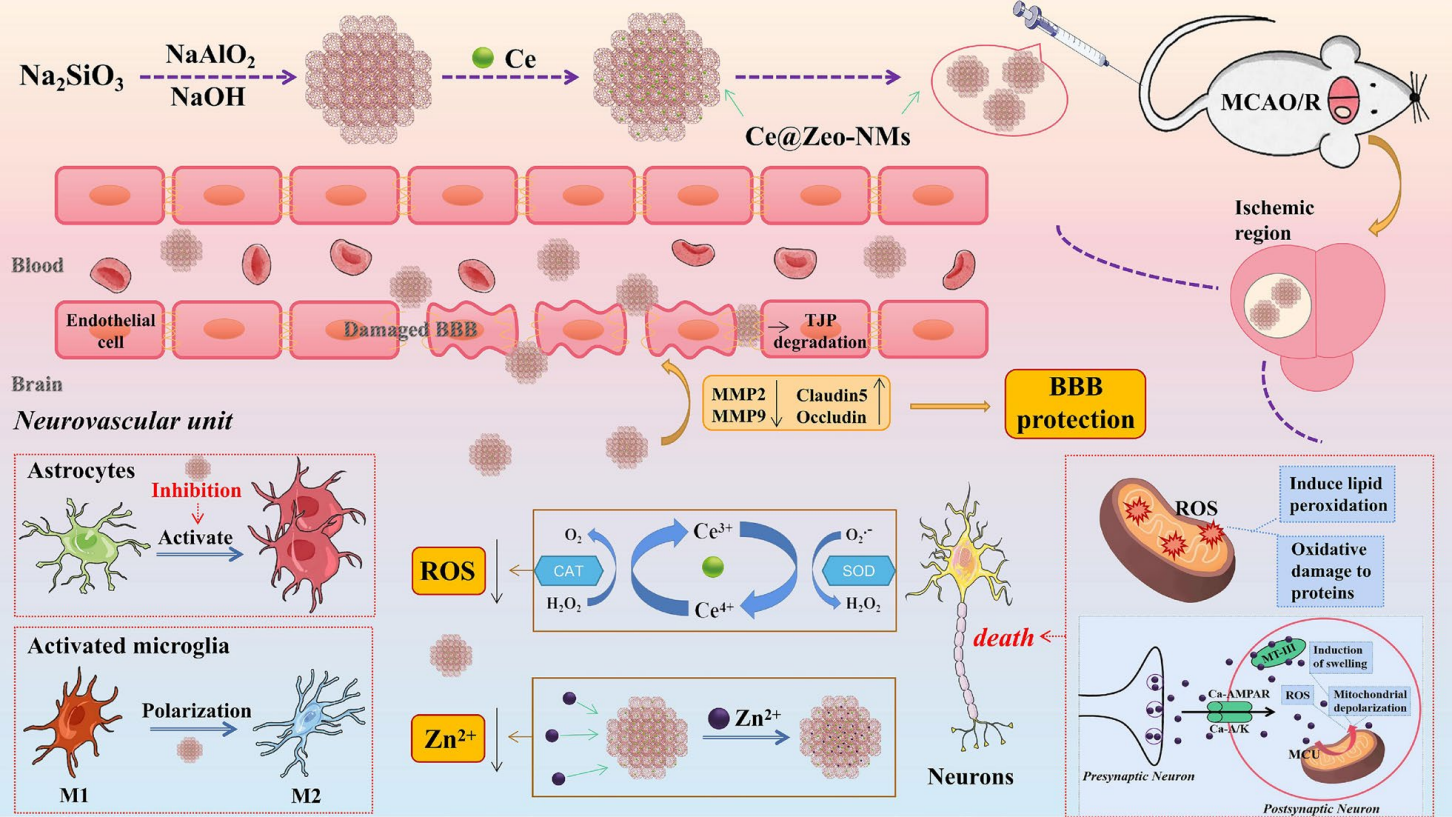
The primary hypothesis of this study. A Ce doped zeolite-based nanoenzyme reduced injury of NVU via efficient removal of Zn^2+^ and ROS [125]. Reproduced with permission copyright 2022, Elsevier

Huang et al. reported the development of cerium (Ce)-doped Linde Type A (LTA) zeolite-based nanomaterials (Ce/Zeo-NMs), showcasing their significant potential in medical applications due to their high porosity, biocompatibility, and biological stability [125]. These nanomaterials are designed as multifunctional mesoporous nanoenzymes to address neurovascular unit (NVU) dysfunction and attenuate cerebral ischemia-reperfusion (I/R) injury. Ce@Zeo-NMs possess a unique adsorption capacity and mimic catalytic activities, enabling them to adsorb excess zinc ions and scavenge ROS generated during acute I/R. This leads to a reshaping of the oxidative and zinc microenvironment in the ischemic brain. *In vivo* studies have demonstrated that Ce@Zeo-NMs effectively reduce ischemic damage in the NVU by decreasing the infarct area, protecting the BBB integrity by inhibiting the degradation of tight junction proteins, and suppressing the activation of microglia and astrocytes in a rat model of middle cerebral artery occlusion-reperfusion (MCAO/R). Specifically developed to induce neuroprotection post-ischemic stroke, these nanomaterials mitigate NVU dysfunction through their ability to absorb excessive Zn^2+^ and mimic enzymatic activities, leading to improved BBB integrity and reduced cerebral ischemia. The study suggests that Ce@Zeo-NMs are a promising multitarget combination therapy with significant neuroprotective effects, offering a new therapeutic strategy for the treatment of ischemic stroke (Fig. 13). This research underscores the potential of Ce-doped zeolite-based NPs in neuroprotection and functional recovery following the onset of ischemic stroke.

### Therapeutic application of nanozymes in Alzheimer’s Disease

Nanozymes possess the capability to penetrate the brain by exploiting the reversibly disrupted BBB, contributing to the maintenance of brain equilibrium in normal physiological circumstances [8]. BBB is made of capillary endothelial cells, astrocytes and pericytes combined to form a protective layer to the brain that can prevent more than 98% of drugs or infective agents from entering the CNS. The ability of BBB recovery over injury happens in a short period of time and controlling the BBB opening is crucial for nanozymes to enter into the brain [126]. Based on reports, Hwang et al. reported that the nanocarriers conjugated with mannitol could help the drug molecules to gain access to the brain through transient BBB opening. Nanocarriers facilitated the transportation of therapeutic drug molecules to the brain, enhancing their efficient delivery [127]. The substantial contribution of nanozymes to the detection and regulation of CNS diseases arises from their catalytic attributes and biocompatibility. The evaluation of nanozyme safety predominantly centers on aspects such as biodistribution, biotoxicity, and pharmacokinetics [63]. Many studies have been conducted and reported the nanozymes potential to enhance the performance in animal models of neurodegenerative diseases, particularly AD [128]. The accumulation of amyloid plaques within brain cells and the formation of neuronal fiber tangles represent two crucial pathological indicators in the progression of AD. Inflammation and the presence of ROS are also key contributors to AD development [129]. Consequently, the current therapeutic approaches predominantly target the reduction of amyloid plaque growth, reduction of inflammation, and ROS elimination.

Oxidative stress-related oxidative damage and inflammation are considered significant contributing factors to brain injury, the aging process, and chronic neurodegenerative conditions. An abnormal surge in ROS levels within cells triggers cellular oxidative stress, leading to DNA harm, inflammatory reactions, cellular senescence, and programmed cell death. Peroxides and nitrogen oxides play a role in various separate mechanisms, such as lipid peroxidation and DNA damage, contributing to the orchestration of oxidative damage and neurotoxic effects. Free radicals can interact with all essential cellular components, leading to lipid peroxidation and the oxidation of DNA and proteins. Lipid peroxidation can bring about membrane impairment through compounds like 4-hydroxynonenal (4-HNE), and the ultimate toxic byproducts adversely impact neurons and the brain's white matter, including axons and oligodendrocytes [130]. Proteins suffering damage and subsequent functional impairment, as well as the activation of DNA repair mechanisms due to peroxidation, result in the swift exhaustion of intracellular energy and culminate in cellular death. In healthy cells, numerous antioxidant enzymes effectively remove surplus ROS and uphold proper mitochondrial function. Nonetheless, within diseased cells, the levels of these enzymes are inadequate to effectively counteract the surplus ROS or, in some cases, these enzymes may not function optimally, leading to an imbalance in cellular redox status. Consequently, the introduction of exogenous antioxidant enzymes becomes imperative to counteract oxidative stress and avert nerve damage [131]. Based on the advanced properties, nanomaterials have been attractive in AD diagnosis and treatment. They are used in research involving AD management, mainly including metal, metal oxide, carbon based, and liposome based NPs are being used in AD diagnosis and treatment.

Tau pathology, characterized by the clustering of tau protein due to excessive phosphorylation as a result of imbalances in the activity of phosphatases and tau kinases, plays a pivotal role in the progression of AD [144]. Elevated levels of hyperphosphorylated tau enhance the likelihood of pathogenic changes in the protein's shape, leading to the formation and clustering of tau fibrils. This issue is distinct from Aβ and is strongly associated with cognitive impairments. Furthermore, tau protein is vital for cellular cargos through the axons, including mitochondria. When this process malfunctions, it triggers oxidative stress and disrupts mitochondrial function [145]. As a result, irregular tau is a significant pathogenic factor in the initiation and progression of AD, emphasizing the value of drug candidates that target tau-related issues. Various attempts have been made to develop drug candidates that address these processes. However, clinical trials demonstrate that treatments focusing solely on one aspect have yielded limited success in AD therapy. This highlights the intricate nature of AD mechanisms and underscores the necessity for comprehensive therapeutic strategies [146]. Researchers, led by Chen et al., introduced a tau-targeted nanocomposite labeled as CeNC/IONC/MSN-T807. With strong affinity the nanocomposite binds with phosphorylated tau and concurrently obstructed several pivotal pathways involved in tau-related AD progression. The study demonstrated that CeNC/IONC/MSN-T807 effectively downregulated glial proliferation, counteracted redox imbalance, reduced hyperphosphorylation of tau, ultimately leading to the enhancement of cognitive behavior of the animals induced with AD. This therapeutic strategy reported the potential for combating synaptic dysfunction and pathological tau accumulation in AD [8].

Elimination of ROS and dissociation of Aβ fibrils are effective therapeutic strategies for correcting the AD microenvironment. Xiaolei Song reported a novel Prussian blue-based nanomaterial (PBK NPs), which addresses the complex pathogenic mechanisms of AD pathology by simultaneously targeting excess ROS and Aβ protein accumulation. These nanomaterials exhibited strong antioxidant activity and responded to NIR light for photothermal effect. Their mechanisms of action mimics SOD, POD, and CAT, effectively reducing ROS levels and oxidative stress. A raise in temperature locally after NPs administration and NIR irradiation, effectively broken the Aβ fibrils down. Incorporation of CKLVFFAED peptide to PBK NPs has eased their entry into the blood-brain barrier and interact with Aβ. *In vivo* results from this study has shown significant reduction in Aβ plaques and neuroinflammation. Neuroprotection by regulation of redox balance and Aβ clearance are primary targets of these nanomaterials which paves ways for many similar multifunctional nanomaterials in AD management. Notably, PBK NPs offer distinct advantages during prevention and treatment of AD such as facile synthetic strategy, combination of ROS and photothermal responsive applications, peptide modification and enhanced BBB entry to carryout Aβ clearance [144].

Cells have been extensively employed in the fabrication of biomimetic nanomaterials to replicate both the physical and chemical attributes of the source cells, and enhance the therapeutic outcomes [147]. Biomimetic materials inspired or imitating erythrocyte membranes offer many beneficial properties such as immunogenicity, prolonged circulation within the bloodstream, and the prevention of protein corona formation [148]. Circulating Aβ peptides binds to the erythrocytic complement receptors (CR1 mediated by C3b complex) and get internalized into hepatocytes for degradation. This report provides the possibility and potential of erythrocyte membrane and pertaining formulations in Aβ and peripheral treatment methods. However, poor specificity of erythrocytes to Aβ is one of the major drawbacks in this strategy, which prompts the feasibility of nanomaterials formulated with erythrocyte membranes for addressing the challenges during AD management. Nanozymes, with antioxidant like activity, exhibit considerable promise in mitigating diseases linked to oxidative stress due to their controlled synthesis, excellent biocompatibility, and robust stability which makes them a viable alternatives to conventional antioxidants or natural enzymes.

**Fig. 14:**
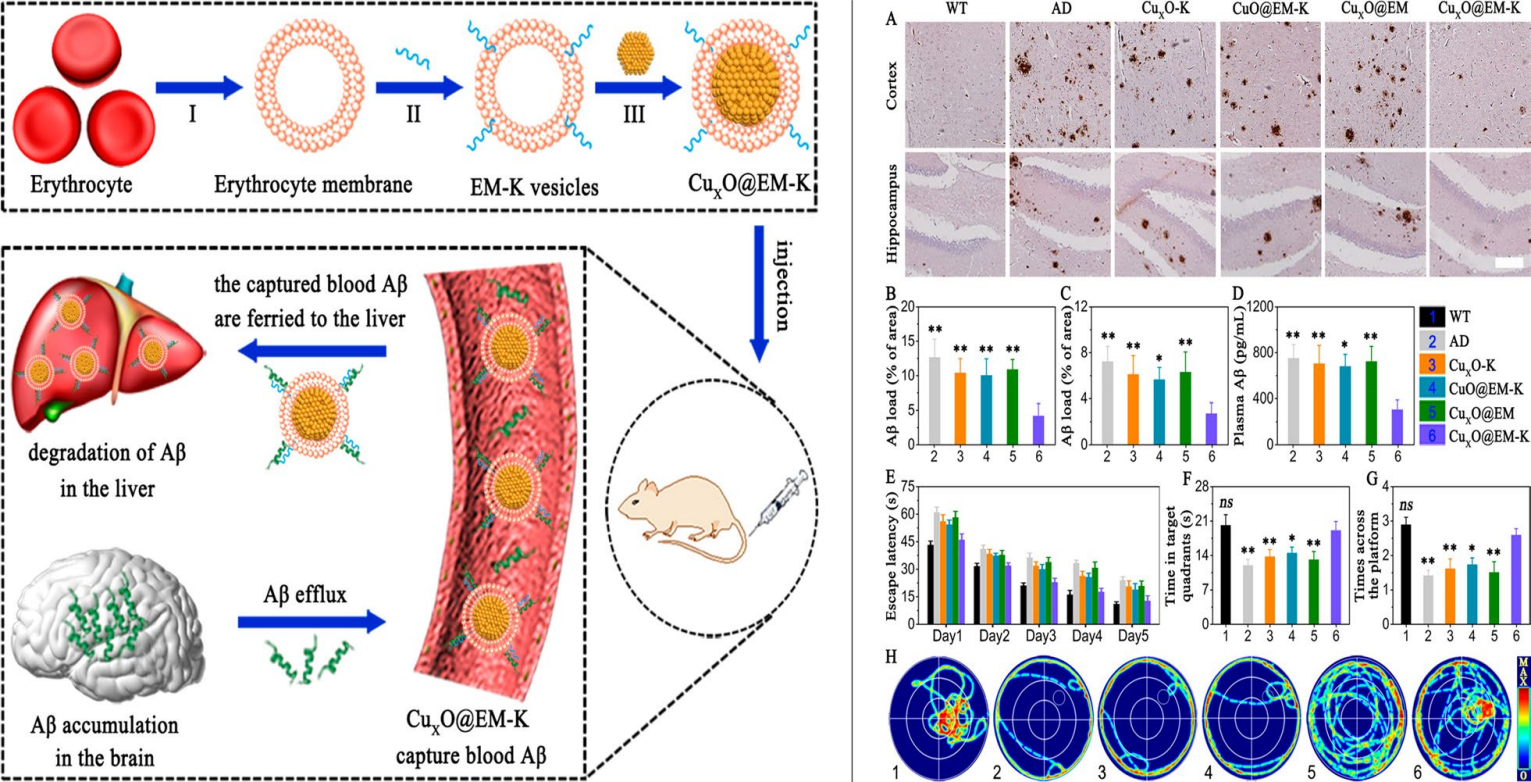
Left panel. Schematic diagrams of CuxO@EM-K synthesis and Peripheral Aβ clearance by CuxO@EM-K. (I) Preparation of erythrocyte membrane vesicles through a hypotonic treatment. (II) Incubation of erythrocyte membrane with Aβ-targeting molecule (DSPE-PEG-K) to prepare EM-K vesicles. (III) Fusion of EM-K vesicles onto CuxO (CuxO@EM-K). The resulting CuxO@EM-K captures Aβ in the blood followed by elimination of Aβ bound to CuxO@EM-K by the liver. Subsequently, clearance of peripheral Aβ facilitates a large efflux of Aβ from the brain into blood through the sink effect, leading to the reduction of brain Aβ burden; Right panel. Reduction of Aβ deposits and amelioration of memory deficits during treatment. **A** Representative images of Aβ staining in both the cortex and the hippocampus. Corresponding quantification of Aβ plaques in the cortex (**B**) and hippocampus (**C**). **D** Blood Aβ levels measured after treatment with CuxO@EM-K. **E** Escape latency to the platform in the training phase. **F** Time spent in the target quadrant. **G** platform entries. **H** Representative swimming paths of mice in the probe test. Data are mean ± SD (n = 6). ns: no statistical difference,*P < 0.05 and **P < 0.01. Reproduced with permission copyright 2020, American Chemical Society [149]

Antioxidant nanozyme developed by Mengmeng Ma and coworkers was incorporated with Aβ-targeting peptide-modified erythrocyte membrane. As the primary objective of the study was to effectively remove peripheral Aβ in AD, CuxO@EM-K was fabricated by enclosing a CuxO nanozyme within a modified 3xTg-AD mouse erythrocyte membrane with the Aβ-targeting peptide KLVFF. KLVFF serves as a “binding element” for targeting Aβ in CuxO@EM-K nanozymes synergistically increasing Aβ concentration in the bloodstream aided by precise targeting with the extended circulation time and low immunogenicity of the erythrocyte membrane. Erythrocyte membrane prevents protein corona formation, thereby preserving Aβ-targeting efficacy of the nanozyme in the bloodstream, while, CuxO core functions as a potent antioxidant enzyme that alleviates Aβ-induced oxidative damage which significantly enhances the erythrocyte membrane’s stability and biomimetic property of CuxO@EM-K helping in synergistic peripheral clearance of Aβ (Fig. 14). This was comprehensively achieved through *in vivo* experiments that substantiated the AD managing ability of CuxO@EM-K's where the nanozyme showed reduction of Aβ levels in both brain and blood with improved cognitive behaviors. In summary, this biomimetic CuxO@EM-K nanozyme, offers a safe and efficient strategy for peripheral Aβ clearance linked to AD [149].

The strategy to counter Aβ-induced neurotoxicity starts with reducing Aβ levels in the brain and decreasing Aβ aggregation. Altering the ratio of Aβ monomers to aggregates without reducing the overall accumulation of Aβ and dosage variations according to the Aβ expression levels among the AD patients might end up with adverse reactions at higher concentrations. Recent research has indicated that the release of Aβ is controlled through breakdown processes by external enzymes such as trypsin, neprilysin (NEP), insulin degrading enzyme (IDE), endothelin-converting enzyme 1 (ECE-1), angiotensin-converting enzyme (ECE-2, ACE), matrix metalloproteinases (MMPs), presequence peptidase (PreP), and plasmin [150]. Exploring the hydrolytic degradation pattern followed by these enzymes for cleaving Aβ peptides, might help in designing an "active treatment" strategy for AD for enhanced efficiency and safety for patients [151]. Chelation therapy using metal ions has been employed to counteract AD progression by preventing Aβ accumulation and scavenging the ROS. Developing a method to exploit these chelating metals beneficially for AD treatment is of utmost importance [152]. Nanomaterials as nanozymes with the potential to function as more precise and efficient "enzymes," have received only limited consideration in the context of AD treatment.

**Fig. 15:**
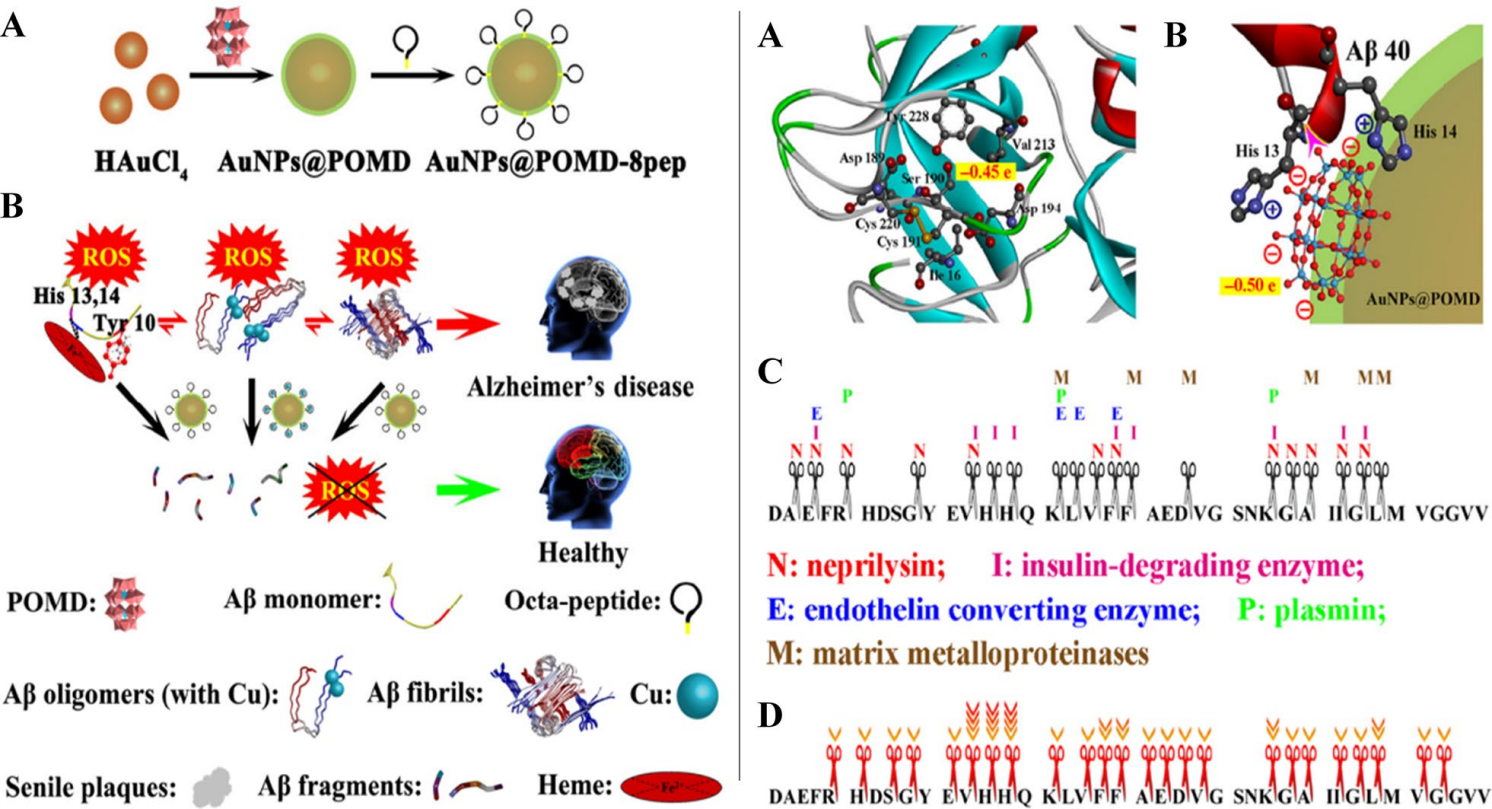
Left panel. Scheme Aβ pathways influenced by AuNPs@POMD-8pep. **A** Synthetic route of the nanozyme. **B** AuNPs@POMD-8pep acted as a multifunctional nanozyme to modulate multiple facets of Alzheimer’s disease; Right panel. Working principle and cleavage sites of AuNPs@POMD8pep on Aβ40. **A** Mulliken charge distributions for the Ser190 hydroxyl group in native protease, and **B** the oxygen groups of POM in the nanozyme. The purple arrow in (**B**) Illustrates the electronegative oxygen groups of POM attacking the electropositive carbon atom in the peptide bond. **C** Cleavage sites of the most important Aβ-degrading enzymes on Aβ40. **D** Cleavage sites of AuNPs@POMD-8pep on Aβ40. More red arrows are assigned to the sites appearing earlier in the analysis. Reproduced with permission copyright 2016, Springer [151]

Nan Gao and colleagues developed Au NPs that can enhance electron transfer and formulated a combined POMD-peptide compound on the surface of the AuNPs, by one step systhesis. According to the simple Au-S bonding, the seven-peptide chain was also conjugated to AuNPs through cysteine residue at the N-terminus. The POMD, AuNPs, and the N-Ac-Cys-heptapeptide (AuNPs@POMD-8pep) complex was designed in order to access easy entry through blood brain barrier and to function as a versatile nanozyme with potential applications in Alzheimer's disease treatment. Modified AuNPs@POMD8pep showed SOD-like activity that was found to be upregulated in the presence of Cu ions and showed dual mode nano-catalytic activity. The AuNPs@POMD-8pep nanozyme exhibited superior effectiveness compared to the natural enzymes in breaking down Aβ aggregates. This enhanced activity of the nanozyme was attributed to the presence of electronegative oxygen groups on the POM's surface. Recent studies have highlighted the crucial role of copper (Cu) in the development of AD by promoting the aggregation of Aβ and generating ROS through the formation of an Aβ/Cu complex. This complex is considered a primary cause of cellular damage due to intermediate products in the fibril formation process, ultimately leading to neuronal death. As a result, there is growing interest in the use of metal-chelating agents like clioquinol (CQ) for treating AD [153]. Considering that the ability of metal ions to aid in AD therapy, AuNPs (AuNPs@POMD-8pep) was presumed to serve as an efficient vehicle for drug delivery across the BBB. The results indicated that the Au-based NPs, AuNPs@POMD-8pep, could cross the BBB as anticipated (Fig. 15) [154].

For a while, fullerenes have been recognized as a nanozyme with SOD-like properties, both *in vitro* and *in vivo*. Qu and colleagues discovered that fullerenes can, generate ROS (superoxide radicals and singlet oxygen) through electron transfer pathways. These fullerenes were modified with active targeting peptides and up-conversion nanoparticles (UCNP) for light-controlled therapy of AD. UCNP transfers energy from NIR region when they were exposed to light, exciting fullerenes to produce ROS. The presence of ROS slows down the aggregation of amyloid-β peptide (Aβ) and mitigates AD progression. The adverse effects of excessive ROS was prevented by antioxidant property of fullerenes without light. Effect of fullerene nanozyme on AD models such as *C. elegans* demonstrated remarkable neuroprotection against Aβ aggregation mediated toxicity. Furthermore, the UCNP in this nanozyme system served as an MRI contrast agent, providing imaging guidance for the treatment process [155].

## Ultrasound triggered nanozymes treat Alzheimer’s disease

Recent investigations have explored the effect of combining FUS and contrast microbubbles (MBs) as an interesting therapeutic strategy for addressing AD by facilitating the opening of the BBB [156]. FUS has been utilized for neurosurgical applications since 1955 and many studies are in support of Low-intensity pulsed focused ultrasound (LIFUP) that has emerged as a safer option, demonstrating reduced damage and the ability to modulate neuronal activity. The utilization of microbubbles in conjunction with ultrasound has proven advantageous in precise diagnosis of tumors, microcirculation, and therapeutic efficacy. Introducing microbubbles before ultrasound application reduces neuronal harm and damaging neurovascular outcomes there by protecting the targeted area. LIFUP utilizes lower energy and pressure, followed by localized cavitation created by microbubble injection, which aides in transient opening of the BBB. This controlled BBB opening facilitates the delivery of immunotherapeutic drugs designed to combat Aβ plaque formation during AD progression. Notably, studies involving animal models such as rodents, rabbits, and monkeys have reported successful applications of FUS with contrast MBs in achieving this objective [157].

A significant hurdle in AD treatment is posed by BBB, lading to considerable challenge for developing effective AD drugs. Recently, Professor Van der Jeugd's studied on brain related diseases highlighted the potential of non-invasive US to enhance the transport of therapeutic antibodies targeting AD into the brain. Additionally, US alone enhanced clearance of tau aggregates and alleviated AD symptoms in mice. Combination of therapeutic strategies, for other brain disorders, has shown higher efficiency than individual approaches. The combination of microbubbles with US-mediated BBB opening for drug delivery has gained attention. These microbubbles, composed of stable air entrapped in lipids, polymers, or proteins layer with 1–5 micron diameter, induce "sonoporation" effects under US irradiation. This mechanism can also involve loading nanoparticles onto the microbubble shell, enabling drug release through ultrasonic pulses. Simultaneously, the "sonoporation" effect enhances cell membrane permeability, facilitating effective drug penetration into cells and ultimately enhancing drug bioavailability. It is an upcoming novel approach to study how cavitation effect of ultrasound induced by microbubbles can be regulated and how to use microbubbles as a drug delivery agent for various CNS related diseases under safe, effective and controllable conditions [158].

**Fig. 16:**
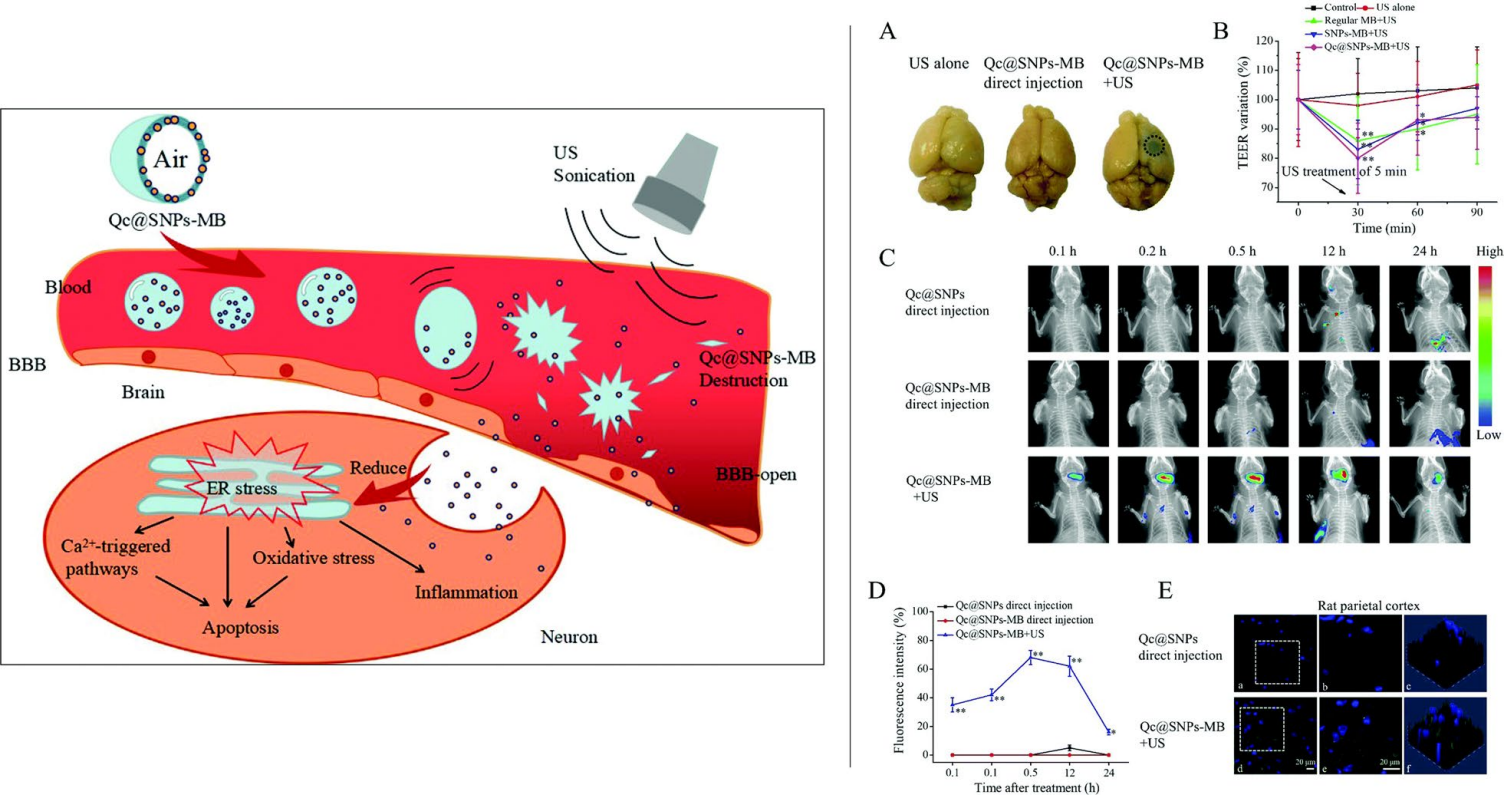
Left panel. Schematic of the present study. In the presence of extracranial US, Qc@SNPs-MB is destroyed to form Qc@SNPs, which has increased BBB permeability. After crossing the BBB, Qc@SNPs is taken up by neurons and reduce the intracellular ER stress, and the apoptosis, inflammation, homeostasis of calcium ions and oxidative stress induced by ER stress; Right panel. BBB permeability of NPs. **A** Opening of the BBB was assessed using Evans blue. **B** Change in the TEER value of the bEnd.3 cell monolayer. **C** *In vivo* fluorescence images of mice given Qc@SNPs by direct injection, Qc@SNPs-MB by direct injection and Qc@SNPS-MB + US. **D** Fluorescence intensity at different times in the brain of the mice. The data are presented as mean ± standard deviation (n = 3). The labels *, **, and *** indicate p < 0.05, p < 0.01, and p < 0.001, respectively, compared with the control group and Qc@SNPs direct inject group. **E** Confocal fluorescence images of the mice parietal cortex. Reproduced with permission copyright 2020, Royal Chemical Society [158]

Yanan Liu conducted a study where they fabricated poly(α-cyanoacrylate n-butyl acrylate)-based microbubbles (Qc@SNPs-MB) with incorporating quercetin conjugated nano-sulfur (Qc@SNPs) onto the surface. which facilitated the transient opening of the BBB and enabled the delivery of NPs. *In vitro* results showed that intracellular entry of Qc@SNPs was aided by US mediated sonoporation, and upon entering the cells they exhibited significant reduction in cellular apoptosis, inflammation, calcium imbalance, and oxidative stress linked to endoplasmic reticulum (ER) stress. *In vivo* study showed that the Qc@SNPs-MB combined with US enabled BBB opening, allowed Qc@SNPs accumulation in the brain, resulting in improved cognitive function in AD mice, increased Aβ clearance, and neuronal preservation. A Morris water maze test indicated that the treatment, involving microbubbles, US, and NPs, did not alter the mice’s behavior or vision. AD induced mice treated with Qc@SNPs-MB+US major improvements in drugs molecules crossing BBB and showes enhanced therapeutic outcomes. The MB formulation triggered with US treatment has reduced Aβ plaques and effectively protected the neuronal cells, indicating potential for AD management (Fig. 16). Notably, Qc@SNPs exhibited superior therapeutic effects compared to SNPs alone. The study’s findings suggested that Qc@SNPs effectively reduced endoplasmic reticulum stress and improved AD-related outcomes due to its target specificity and biocompatibility. This nanoformulation offered a promising alternative therapeutic strategy for neurodegenerative disease, specifically addressing drug delivery challenges across the BBB. The sonoporation effect induced by MB and US, showed major impact in research pertaining to BBB permeability and effective drug delivery to the central nervous system [158].

## Conclusion and prospectives

Nanozymes have garnered significant attention due to their superior catalytic abilities and resilience in harsh conditions, making them promising alternatives to natural enzymes in various fields. They are particularly remarkable in biomedical applications, where their physicochemical properties allow them to adapt to different environments. Factors like pH, redox conditions, and oxygen levels can influence their catalytic behavior. Nanozymes can also be stimulated by external energy sources like heat, light, magnetic fields, and US. This review highlights the importance of US-responsive activity and controlled release in maximizing nanozymes therapeutic impact while minimizing side effects. Nanozymes have led to innovation in biomedicine therapeutic areas of cancer treatment, wound healing, neurology (including AD and PD), and biosensing. We have summarized the most recent advancements in research and approaches for creating potential therapeutic nanozymes aimed at addressing neurological disorders. AD is one of the challenging neurodegenerative diseases and recent progress in designing nanozymes has paved way for AD treatment. The existing approach involving FDA-approved drugs have limitations due to physiological factors and disease complexity. Nanozymes offer a potential solution by enabling targeted drug delivery to the brain. The challenges of AD therapy that are exacerbated by drug side effects, can be addressed effectively through nanomedicine approach. Nanocarriers, with their immunogenicity, biocompatibility, size, and degradability, hold promise for delivering hydrophilic and hydrophobic drugs across BBB. However, nanomedicines for AD treatment, need significant advancement to fabricate them as potent, and safe formulations to overcome the challenges posed by this disease.

Recently, US based methods have gained attention for their potential to enhance drug delivery across BBB. Certain nanozymes that respond to external stimuli, such as laser, US, and magnetic fields, can be activated for drug release and therapy. US is an effective external stimulus that has gained rapid development due to its precise control, non-invasiveness, tissue-penetrating capability, convenience, and affordability. This sonodynamic technique employs MB, small gas/ or air filled vesicles stabilized by lipids, polymers, or proteins to enhance the permeability of vascular and cellular membranes. Microbubbles also serve as contrast agents for US imaging and regulate the acoustic forces needed for sonoporation. In addition to permeabilizing membranes, microbubbles have been used for thrombolysis and direct drug delivery. When loaded with therapeutic agents, they can release these agents upon exposure to destructive US pulses. Recent studies have shown that non-invasive ultrasound can improve the transport of therapeutic antibodies targeting AD pathogenic signaling in the brain and even alleviate symptoms in mice. Nanozymes with multiple stimuli-responsive properties were successfully developed for addressing limitations of single stimulus-responsive nanozymes. However, the increased complexity also poses challenges in regulating catalytic activities. Nanozymes and ultrasound therapy have shown great promise in treating various applications such as tumors, infection/antimicrobial resistance, wound healing, biosensing, imaging, various neurological diseases. There's growing interest in using US-triggered nanozymes for neurological therapies with minimal side effects. Current focus of the nanotechnologists and clinicians are on establishing US-triggered nanozymes as a safer and more effective AD theragnostic platform, however, extrnsive research is required for reducing the challenges faced during fabrication of nanozymes and exection of nanocatalytic therapy for improved safety and efficacy.

Nanozymes, with their enzyme-like activities, offer several advantages in AD therapeutics compared to traditional approaches. Their ability to target specific brain regions or cells enables precise drug delivery, reducing off-target effects and enhancing efficacy. A significant benefit of nanozymes is their capacity to penetrate the BBB efficiently, a crucial factor in delivering drugs to the brain areas affected by AD. Targeted delivery contributes to reduced side effects, improving patient compliance and minimizing adverse reactions. Nanozymes also exhibit enhanced stability, ensuring longer circulation time and improved drug bioavailability. Their multifunctionality is another advantage, as they can simultaneously act as drug carriers, imaging agents, and therapeutic agents, offering a comprehensive approach to AD treatment. Nanozymes can mimic the activity of natural enzymes like catalase and peroxidase, neutralizing ROS and reducing oxidative stress associated with AD progression. Their design often focuses on biocompatibility, which is essential for safe therapeutic interventions. Additionally, some nanozymes can function as imaging agents, aiding in the visualization of amyloid plaques and facilitating both diagnosis and treatment monitoring. Customization and tunability of nanozymes allow them to be tailored for catering individual patient needs or specific disease subtypes, which significantly enhances the treatment precision. Despite their potential, it's important to note that nanozyme research for AD therapy is still in early stages, and ongoing research is crucial to address existing challenges and optimize nanozyme design for effective treatment strategies.

Advancements in the field of nanozymes for AD treatment have shown significant progress, including the use of ultrasound to release therapeutic agents from nanozymes within specific affected brain areas, greatly enhancing drug delivery precision and efficiency. Innovations in nanocatalysis have led to improved nanozymes, particularly in addressing Alzheimer's pathological features like beta-amyloid plaque degradation. Nanozymes have been tailored as therapeutic carriers activated by ultrasound, ensuring controlled and localized therapeutic release. Future research will prioritize multifunctional nanozymes that can target various aspects of Alzheimer’s pathology by combining catalytic activity with functionalities like biomolecule targeting or imaging. Further developments in ultrasound technologies aim to enhance precision and safety in nanozyme activation, potentially improving control over drug release and therapeutic effectiveness. Overcoming the blood-brain barrier penetration challenge will remain as a significant research focus for precise brain targeting. The field may advance toward personalized treatments, customizing ultrasound-triggered nanozymes based on individual patient profiles and Alzheimer’s disease variations for improved treatment outcomes. Additionally, exploring synergistic effects by combining ultrasound-triggered nanozymes with other therapies, such as immunotherapies or traditional drugs, holds potential for a more comprehensive and effective approach to Alzheimer’s treatment.

The clinical application of USRN (Ultrasound-Responsive nanozymes) for treating neurological diseases necessitates a thorough assessment of their long-term safety. The fusion of brain nanomedicine and US technology aims to achieve comprehensive cell analysis in a laboratory setting and conduct initial assessments in living animals. Nevertheless, replicating the intricate structure of the human brain poses challenges when using experimental animals. For instance, notable distinctions exist between rodents and humans in terms of brain size, brain tissue morphology, and certain protective features such as the skull and the BBB [29]. While numerous studies in mouse models have reported positive safety results thus far, it is essential to acknowledge that data from mouse studies alone are insufficient for clinical translation [159]. Moreover, most of the safety data gathered so far has been limited to short-term assessments. The absence of long-term safety data underscores the importance of conducting both preclinical and clinical studies to evaluate the compatibility of USINs before their use in human patients. Given the delicate nature of the brain, it is crucial to implement standardized and rigorous evaluation methods to ensure the generation of highly reliable data for clinical implementation. These evaluations are vital for identifying potential adverse effects and developing materials that are safer and more effective [28]. In summary, there is a need to intensify research efforts to delve into the composition and technological advancements of USRN. Additionally, comprehensive research into the causes, treatment options, and rehabilitation mechanisms of neurological diseases is essential. Simultaneously, improvements in ultrasound equipment that can work seamlessly with USRN should be prioritized. Undoubtedly, considering the current research demonstrating the unique advantages of USRN in treating neurological diseases, we are optimistic that as this emerging field continues to develop, this technology will find more effective applications in biomedicine, further enhancing human health.

## Data Availability

No data was used for the research described in the article.
